# Supplementary material for: Osteology of Galeamopus pabsti sp. nov. (Sauropoda: Diplodocidae), with implications for neurocentral closure timing, and the cervico-dorsal transition in diplodocids
Source: PeerJ. 2017 May 2;5:e3179. doi: 10.7717/peerj.3179 (PMC5417106; doi:10.7717/peerj.3179)
Supplement: Data S1 — Modified from Tschopp, Mateus & Benson (2015a)) [file peerj-05-3179-s001.docx]

1. Premaxilla, anterior margin, step-like transition to nasal process: absent (0); present (1).

(Upchurch 1998 (C10); Wilson & Sereno 1998 (C19); wording modified)

2. Premaxilla, anterior margin, step-like transition to nasal process, position of inflection point in lateral view: close to subnarial foramen (0); significantly posterior to subnarial foramen (1).

(Carballido et al. 2012 (C2); modified)

3. Premaxilla, external surface: without anteroventrally orientated vascular grooves originating from an opening in the maxillary contact (0); vascular grooves present (1).

(Wilson 2002; Sereno et al. 2007)

4. Premaxilla, shape in dorsal view: main body massive, with proportionally short ascending process distinct (0); single elongate unit, distinction between body and process nearly absent (1).

(Upchurch 1998; modified by Tschopp et al. 2015)

5. Premaxilla, angle between lateral and medial margins of premaxilla as seen in dorsal view: > 40° (0); 17°-40° (1); < 17° (2).

(Upchurch 1999, modified Tschopp et al. 2015)

6. Premaxilla, posteroventral edge of ascending process, shape in lateral view: concave (0); straight (1).

(Whitlock 2011, wording modified by Tschopp et al. 2015)

7. Premaxilla, posteroventral edge of ascending process, orientation of ventral-most portion: vertical (0); posterodorsally inclined (1).

(Tschopp et al. 2015)

8. Premaxilla, posterolateral process and the lateral process of the maxillary, shape: without midline contact (0); with midline contact forming a marked narial depression, subnarial foramen not visible laterally (1).

(Wilson & Sereno 1998)

9. Premaxilla, dorsoventral depth of anterior portion: remains the same as posteriorly, or widens gradually (0); widens considerably, and abruptly (1).

(Harris 2006a)

10. Subnarial foramen and anterior maxillary foramen, position: well distanced from one another (0); separated by narrow bony isthmus (1).

(Wilson 2002)

11. Maxilla, large foramen anterior to the preantorbital fossa, separated by a narrow bony bridge: absent (0); present (1).

(Zaher et al., 2011)

12. Maxilla, large foramen posterior to anterior maxillary foramen, dorsal to preantorbital fossa: absent (0); present (1).

(Tschopp et al. 2015)

13. Anterior maxillary foramen, location: detached from maxillary-premaxillary boundary, facing dorsally (0); lies on medial edge of maxilla, opening medially into the premaxillary-maxillary boundary (1).

(Tschopp et al. 2015)

14. Maxilla, canal connecting the antorbital fenestra and the preantorbital fossa: absent (0); present (1).

(Tschopp et al. 2015)

15. Maxilla, dorsal process, posterior extent: anterior to or even with posterior process (0); extending posterior to posterior process (1).

(Whitlock 2011, wording modified by Tschopp et al. 2015)

16. Maxilla-quadratojugal contact: absent or small (0); broad (1).

(Yu 1993)

17. Preantorbital fossa: absent (0); present (1).

(Tschopp & Mateus 2013)

18. Preantorbital fossa, if present: with relatively indistinct borders (0); dorsally capped by a thin, distinct crest (1).

(Wilson 2002, modified by Tschopp et al. 2015)

19. Preantorbital fenestra: reduced to absent (0); present, occupying at least 50% of the preantorbital fossa (1).

((Berman & McIntosh 1978), Tschopp et al. 2015)

20. Antorbital fenestra, maximum diameter: much shorter (<90%) than orbital maximum diameter (0); subequal (>90%) to orbital maximum diameter (1).

(Yu 1993; Whitlock (2011) defined a modified quantitative state boundary, which was changed from 85% to 90% by Tschopp et al. 2015)

21. Antorbital fenestra, anterior extension: is restricted posterior to preantorbital fossa (0); reaches dorsal to preantorbital fossa (1).

(Tschopp et al. 2015)

22. Antorbital fenestra, shape of dorsal margin: straight or convex (0); concave (1).

(Whitlock 2011)

23. External nares, position: retracted to level of orbit, facing laterally (0); retracted to position between orbits, facing dorsally or dorsolaterally (1).

(McIntosh 1989; Upchurch 1995; modified by Whitlock (2011))

24. External nares, maximum diameter: shorter than orbital maximum diameter (0); longer than orbital maximum diameter (1).

(Upchurch 1995; state definitions modified by Wilson & Sereno (1998))

25. Prefrontal, medial margin, shape: without distinct anteromedial projection (0); curving distinctly medially anteriorly to embrace the anterolateral corner of the frontal (1).

(Tschopp et al. 2015)

26. Prefrontal, posterior process size: small, not projecting far posterior of frontal-nasal suture (0); elongate, approaching parietal (1).

(Wilson 2002)

27. Prefrontal, posterior process shape: flat (0); hooked (1).

(Wilson 2002)

28. Frontals, midline contact (symphysis): patent suture (0); fused in adult individuals (1).

(Salgado & Calvo 1992; Yu 1993)

29. Frontal, anteroposterior length: long, > 1.4 times minimum transverse width (0); short, 1.4 or less times minimum transverse width (1).

(Whitlock 2011, modified by Tschopp et al. 2015)

30. Frontal-nasal suture, shape: flat or slightly bowed anteriorly (0); v-shaped, pointing posteriorly (1).

(Whitlock 2011)

31. Frontals, distinct anterior notch medially between the two elements: absent (0); present (1).

(Tschopp & Mateus 2013, modified by Tschopp et al. 2015)

32. Frontals, dorsal surface: without paired grooves facing anterodorsally (0); grooves present, extend on to nasal (1).

(Whitlock 2011)

33. Frontal, lateral edge in dorsal view: relatively straight (0); deeply concave (1).

(Tschopp et al. 2015)

34. Frontal, contribution to dorsal margin of orbit: less than 1.5 times the contribution of prefrontal (0); at least 1.5 times the contribution of prefrontal (1).

(Whitlock 2011, modified by Mannion et al. 2012)

35. Frontal, free lateral margin: rugose (0); smooth (1).

(Tschopp & Mateus 2013)

36. Frontal, contribution to margin of supratemporal fenestra/fossa: present (0); absent, frontal excluded from anterior margin of fenestra/fossa (1).

(Wilson & Sereno 1998)

37. Frontal-parietal suture, position of medial portion: closer to anterior extension of supratemporal fenestra (0); closer to posterior extension (1).

(Tschopp & Mateus 2013, modified by Tschopp et al. 2015)

38. Pineal (parietal) foramen between frontals and parietals: present (0); absent (1).

(Yu 1993, modified by Tschopp et al. 2015)

39. Orbit, anterior-most point: anterior to the anterior extremity of lateral temporal fenestra (0); roughly even with or posterior to anterior extent of lateral temporal fenestra (1).

(Gauthier 1986; Upchurch 1995, modified by Whitlock 2011)

40. Orbital ventral margin, anteroposterior length: broad, with subcircular orbital margin (0); reduced, with acute orbital margin (1).

(Wilson & Sereno 1998)

41. Postorbital, posterior process: present (0); absent (1).

(Wilson 2002)

42. Jugal, contribution to antorbital fenestra: very reduced or absent (0); large, bordering approximately one-third of its perimeter (1).

((Berman & McIntosh 1978) Upchurch 1995, modified by Whitlock (2011), who added quantitative state boundary definition)

43. Jugal, contact with ectopterygoid: present (0); absent (1).

(Upchurch 1995)

44. Jugal, posteroventral process: short and broad (0); narrow and elongate (1).

(Tschopp et al., 2015)

45. Jugal, dorsal process: present (0); absent (1).

(Yu 1993)

46. Jugal, anterior spur dorsally, which projects into antorbital fenestra: absent (0); present (1).

(Tschopp et al., 2015)

47. Quadratojugal, position of anterior terminus: posterior to middle of orbit (0); anterior margin of orbit or beyond (1).

(Whitlock 2011)

48. Quadratojugal, angle between anterior and dorsal processes: less than or equal to 90°, so that the quadrate shaft is directed dorsally (0); greater than 90°, approaching 130°, so that the quadrate shaft slants posterodorsally (1).

(Gauthier 1986; Upchurch 1995)

49. Lacrimal, anterior process: absent (0); present (1).

(Wilson 2002)

50. Lacrimal, dorsal portion of lateral edge, eminence: absent (0); present (1).

(Tschopp & Mateus, 2013 (C34); modified)

51. Lacrimal, dorsal portion of lateral edge, eminence, shape: shallow bulge (0); laterally projecting spur (1).

(Tschopp & Mateus, 2013 (C34); modified)

52. Quadrate, articular surface shape: quadrangular in ventral view, orientated transversely (0); roughly triangular in shape (1); thin, crescent-shaped surface with anteriorly directed medial process (2).

(Whitlock 2011)

53. Quadrate, short transverse ridge medially on posterior side of ventral ramus, close to the articular surface with the lower jaw: absent (0); present (1).

(Tschopp et al., 2015)

54. Quadrate fossa, depth: shallow (0); deeply invaginated (1).

(Russell & Zheng 1993)

55. Quadrate, shallow, second fossa medial to pterygoid flange on quadrate shaft (not the quadrate fossa): absent (0); present, becoming deeper towards its anterior end (1).

(Tschopp & Mateus 2013, wording modified by Tschopp et al., 2015)

56. Quadrate, dorsal margin: concave, such that pterygoid flange is distinct from quadrate shaft (0); straight, without clear distinction of posterior extension of pterygoid flange (1).

(Tschopp et al., 2015)

57. Quadrate, posterior end (posterior to posterior-most extension of pterygoid ramus): short and robust (0); elongate and slender (1).

(Tschopp et al., 2015)

58. Squamosal, anterior extent: restricted to postorbital region (0); extends well past posterior margin of orbit (1); extends beyond anterior margin of orbit (2).

(Whitlock 2011)

59. Squamosal-quadratojugal contact: present (0); absent (1).

(Upchurch 1995)

60. Squamosal, posteroventral margin: smooth, or with short and blunt ventral projection (0); with prominent, ventrally directed 'prong' (1).

(Whitlock 2011; modified by Tschopp et al., 2015)

61. Squamosal, posteroventral margin: smooth, without ventral projection (0); ventral projection present (1).

(Tschopp et al., 2015)

62. Parietal, contribution to post-temporal fenestra: present (0); absent (1).

(Wilson 2002)

63. Parietal, portion contributing to skull roof, anteroposterior length/transverse width: wide, > 50% (0); narrow, 7-50% (1); practically inexistant, < 7% (2).

(Tschopp et al., 2015)

64. Parietal, distance separating supratemporal fenestrae: less than 1.5 times the width of the long axis of the supratemporal fenestra (0); at least 1.5 times the length of the long axis of the supratemporal fenestra (1).

(Wilson 2002, modified by Mannion et al. 2012)

65. Parietal, posterolateral process, dorsal edge in posterior view: straight, and ventrolaterally oriented, so that the supratemporal fenestra is slightly facing posteriorly as well (0); convex, so that the postorbital and thus the supratemporal fenestra are not visible (1).

(Tschopp & Mateus 2013)

66. Parietal, occipital process, dorsoventral height: low, subequal to less than the diameter of the foramen magnum (0); high, nearly twice the diameter of the foramen magnum (1).

(Wilson 2002, modified by Tschopp et al., 2015)

67. Parietal, occipital process, distal end: ventrolaterally oriented, such that dorsolateral edge is straight or convex (0); curving laterally, such that dorsolateral edge becomes concave distally (1).

(Tschopp et al., 2015)

68. Parietal, distinct horizontal ridge separating dorsal from posterior portion: absent, transition more or less confluent (0); present, creating a distinct nuchal fossa below the ridge (1).

(Tschopp & Mateus 2013)

69. Postparietal foramen: absent (0); present (1).

(Upchurch 1995)

70. Paroccipital process (popr), posterior face: smooth/flat (0); with longitudinal ridge along popr body extending from dorsomedial to ventrolateral corners (1).

(Tschopp & Mateus 2013)

71. Paroccipital process distal terminus: expanded vertically (0); not expanded (dorsal and ventral edges are subparallel) (1).

(Upchurch 1998, modified by Tschopp et al., 2015)

72. Paroccipital process, distal end in lateral view: straight (0); curved (1).

(Tschopp et al., 2015)

73. Supratemporal fenestra: present, relatively large (anteroposterior diameter is at least 5% of occiput width) (0); absent, or greatly reduced (so that anteroposterior diameter is less than 5% of occipital width) (1).

(Wilson 2002, modified by Mannion et al. 2012)

74. Supratemporal fenestra, maximum diameter: more than 1.2 times greatest diameter of foramen magnum (0); less than 1.2 times the greatest length of foramen magnum (1).

(Yu 1993, modified by Mannion et al. 2012)

75. Supraoccipital, anterodorsal margin: internally concave, associated with a channel extending ventrally on the internal face (0); straight (1).

(Remes 2006)

76. Supraoccipital, dorsal extension: high and vaulted, such that the dorsolateral edges are strongly sinuous (0); low, with the dorsolateral edges straight (1).

(Remes 2006)

77. Supraoccipital: sagittal nuchal crest: broad, weakly developed (0); narrow, sharp, and distinct (1).

(Whitlock 2011)

78. Supraoccipital, foramen close to contact with parietal: absent (0); present (1).

(Tschopp & Mateus 2013)

79. Crista prootica, size: rudimentary (0); expanded laterally into dorsolateral process (1).

(Salgado & Calvo 1992; Upchurch 1995)

80. Occipital condyle, articular surface: well offset from condylar neck (0); continuously grading into condylar neck (1).

(Tschopp et al., 2015)

81. Basioccipital, contribution to dorsal side of occipital condylar neck: present and broad, around 1/3 of entire dorsal side (0); reduced to absent (1).

(Harris & Dodson 2004)

82. Basioccipital, distance from base of occipital condyle to base of basal tubera (best visible in lateral view): short, such that area is gently U-shaped in lateral view (0); elongate, with a flat portion between occipital condyle and basal tubera (1).

(Tschopp & Mateus 2013)

83. Basioccipital depression between foramen magnum and basal tubera: absent (0); present (1).

(Wilson 2002)

84. Basioccipital, pit between occipital condyle and basal tubera: absent (0); present (1).

(Mannion et al. (2013: C98), wording modified by Tschopp et al., 2015)

85. Basal tubera: globular (0); box-like (1).

(Whitlock et al.2010)

86. Basal tubera, breadth: <1.3 times (0); 1.3-1.85 times (1); >1.85 times occipital condyle width (2).

(Wilson 2002, modified by Tschopp et al., 2015)

87. Basal tubera: distinct from basipterygoid (0); reduced to slight swelling on ventral surface of basipterygoid (1).

(Whitlock 2011)

88. Basal tubera, shape of posterior face: convex (0); flat (1); slightly concave (2).

(Whitlock 2011, modified by Tschopp et al., 2015)

89. Basal tubera, posteroventral face: continuous (0); marked by a distinct transverse ridge (1).

(Tschopp et al., 2015)

90. Basal tubera, longest axes: parallel (0); in an angle to each other, pointing towards the occipital condyle (1).

(Tschopp et al., 2015)

91. Basal tubera, anterior edge: straight or convex (0); concave (1).

(Tschopp et al., 2015)

92. Basal tubera in posterior view: facing ventrolaterally (0); facing straight ventrally, forming a horizontal line (1).

(Tschopp & Mateus 2013, wording modified by Tschopp et al., 2015)

93. Basal tubera, foramen in notch that separates the two tubera: absent (0); present (1).

(Tschopp & Mateus 2013)

94. Basisphenoid/basipterygoid recess: absent (0); present (1).

(Wilson 2002)

95. Basipterygoid processes: widely diverging (> 60°) (0); intermediate, 31°-60° (1); narrowly diverging (< 31°) (2).

(Yu 1993, modified by Tschopp et al., 2015)

96. Basipterygoid processes, orientation: directed more than 75° to skull roof (normally perpendicular) (0); angled less than 75° to skull roof (normally approximately 45°) (1).

(McIntosh 1990a, modified by Mannion et al. 2012)

97. Basipterygoid processes, ratio of length:basal transverse diameter: < 4 (0); = or > 4.0 (1).

(Harris 2006c)

98. Basipterygoid, area between the basipterygoid processes and parasphenoid rostrum: is a mildly concave subtriangular region (0); forms a deep slot-like cavity that passes posteriorly between the bases of the basipterygoid processes (1).

(Upchurch 1995, 1998)

99. Basipterygoid processes, orientation of proximal-most portions: same as central portion of shaft (0); parallel to each other, outwards curve of shaft happens only more anteriorly (1).

(Tschopp et al., 2015)

100. Basipterygoid processes, distal end in anterior view: straight (0); curving laterally (1).

(Tschopp et al., 2015)

101. Basipterygoid processes, distal lateral expansion: absent (0); present (1).

(Tschopp et al., 2015)

102. Parasphenoid rostrum, groove on dorsal edge: absent (0); present (1).

(Upchurch 1998, modified by Tschopp et al., 2015)

103. Optic foramen: paired (0); unpaired (1).

((Berman & McIntosh 1978); Sander et al. 2006)

104. Palatobasal contact, shape: pterygoid with small facet (0); dorsomedially orientated hook (1).

(Wilson & Sereno 1998; Wilson 2002)

105. Pterygoid, transverse flange (i.e. ectopterygoid process) position: between orbit and antorbital fenestra (0); anterior to antorbital fenestra (1).

(Upchurch 1995)

106. Vomer, anterior articulation: premaxilla (0); maxilla (1).

(Wilson 2002, polarity inversed by Tschopp et al., 2015)

107. Dentary, anteroventral margin shape: gently rounded (0); sharply projecting triangular process or 'chin' (1).

(Upchurch 1998)

108. Dentary, cross-sectional shape of symphysis: oblong or rectangular (0); subtriangular, tapering sharply towards ventral extreme (1); subcircular (2).

(Whitlock 2011)

109. Dentary, tuberosity on labial surface near symphysis: absent (0); present (1).

(Whitlock 2011)

110. Dentary, anterolateral corner: not expanded laterally beyond mandibular ramus (0); expanded beyond lateral mandibular ramus (1).

(Whitlock 2011)

111. Mandible, coronoid eminence: strongly expressed, clearly rising above plane of dentigerous portion (0); absent (1).

(Whitlock 2011)

112. Surangular foramen: absent (0); present (1).

(Tschopp et al., 2015)

113. External mandibular fenestra: present (0); absent (1).

(Russell & Zheng 1993)

114. Snout shape in dorsal view: premaxilla-maxilla index (PMI; Whitlock et al., 2010) < 67% (0); 67-85% (1); > 85% (2).

(Upchurch 1998, modified by Whitlock 2011, introduced more character states by Tschopp et al., 2015)

115. Shape of tooth row in occlusal view: follows curvature of dentary (0); anterolateral corner of tooth row displaced labially (1).

(Whitlock & Harris 2010)

116. Tooth rows, length: restricted anterior to orbit (0); restricted anterior to antorbital fenestra (1); restricted anterior to subnarial foramen (2).

(Gauthier 1986, modified by Whitlock 2011)

117. Dentary teeth, number: greater than 17 (0); 10-17 (1); 9 or fewer (2).

(Russell & Zheng 1993, modified by Carballido et al. 2012)

118. Replacement teeth per alveolus, number: three or fewer (0); four or more (1).

(Wilson 2002)

119. Teeth, crown-to-crown occlusion: present (0); absent (1).

(Wilson & Sereno 1998; polarity reversed by Whitlock 2011)

120. Teeth, wear facets shape: v-shaped (0); planar (1).

(Wilson & Sereno 1998, modified by Tschopp et al., 2015)

121. Teeth, occlusal pattern: paired wear facets (0); single wear facet (1).

(Wilson & Sereno 1998, modified by Tschopp et al., 2015)

122. Teeth, SI values for tooth crowns: < 3.4 (0); 3.4 or greater (1).

(McIntosh 1989, Upchurch 1998, modified by Tschopp et al., 2015)

123. Tooth crowns, orientation: aligned slightly anterolingually, tooth crowns overlap (0); aligned along jaw axis, crowns do not overlap (1).

(Wilson & Sereno 1998; polarity reversed by Whitlock 2011)

124. Tooth crowns, cross-sectional shape at midcrown: D-shaped (0); cylindrical (1).

(Russell & Zheng 1993, modified by Wilson & Sereno 1998)

125. Teeth, orientation relative to long axis of jaw: perpendicular (0); oriented anteriorly (procumbent) (1).

(Upchurch 1998)

126. Teeth, longitudinal grooves on lingual aspect: absent (0); present (1).

(Wilson 2002)

127. Teeth, thickness of enamel asymmetric labiolingually: absent (0); present (1).

(Whitlock 2011)

128. Teeth, marginal denticles: present (0); absent (1).

(McIntosh 1990a)

129. Presacral neural spines, bifurcation: absent (0); present (1).

((McIntosh 1989); Wilson 2002, modified by Tschopp et al., 2015)

130. Number of cervical vertebrae: < 13 (0); 14-15 (1); 16 or more (2).

(McIntosh 1990a, modified by Tschopp et al., 2015)

131. Cervical vertebrae width to height ratio: less than 0.5 (0); 0.5-1.5 (1); more than 1.5 (2).

(Upchurch et al. 2004b (C1), modified by Tschopp et al., 2015 (C128))

132. Cervical pneumatopores (pleurocoels): absent (0); present (1).

(McIntosh 1990a; Upchurch 1995)

133. Cervical centra, internal pneumaticity: absent (0); present (1).

((Cope 1877); Upchurch 1998; Wilson & Sereno 1998; Wedel 2000, 2002; modified by Carballido et al. 2012; wording modified)

134. Cervical centra, internal pneumaticity, development: simple, few large cavities (0); complex, numerous small, complex cavities (1).

(Wilson & Sereno 1998 (C102); modified after Carballido et al. 2012b (C120))

135. Cervical vertebrae, small fossa on posteroventral corner: absent (0); shallow, anteroposteriorly elongate fossa present, posteroventral to pleurocoel (1).

(Whitlock 2011)

136. Cervical centra, midline keels on ventral surface: prominent and plate-like (0); reduced to low ridges (1).

(Upchurch 1998; Upchurch et al. 2004a)

137. Cervical vertebrae, longitudinal sulcus on ventral surface: absent (0); present (1).

(Upchurch 1995, 1998)

138. Cervical vertebra, posterior projection on transverse processes: present (0); absent (1).

(Remes et al. 2009)

139. Cervical vertebrae, posterior extension of posterior centrodiapophyseal lamina: is anteriorly restricted (0); reaches below posterior end of neural canal (1).

(Tschopp et al., 2015)

140. Cervical vertebrae, short second posterior centrodiapophyseal lamina ventral to the one uniting with the dorsal shelf of the diapophysis: absent (0); present (1).

(Tschopp et al., 2015)

141. Cervical vertebrae, foramen on dorsal side of postzygodiapophyseal lamina, just anterior to base of neural spine process: absent (0); present (1).

(Remes 2007)

142. Cervical vertebrae, epipophysis: reduced or absent (0); pronounced, forming a distinct projection above the postzygapophysis (1).

(Remes et al. 2009, modified by Tschopp et al., 2015)

143. Cervical vertebrae, pneumatized epipophyses: absent (0); present (1).

(Tschopp et al., 2015)

144. Cervical neural spines, bifurcation, if present, anterior extension within column includes: CV 3 (0); all mCV (1); posterior mCV (2); only pCV (3).

(Russell & Zheng 1993, modified by Tschopp et al., 2015)

145. Cervical vertebrae, unbifurcated neural spines in anterior/posterior view: with parallel lateral edges or converging (0); distal end expanded laterally (1).

(Tschopp et al., 2015)

146. Cervical vertebrae, summits of bifid neural spines: are laterally compressed (0); are rounded (1).

(Upchurch et al. 2004b)

147. Proatlas, distal end: broadly rounded (0); narrow, almost pointed (1).

(Tschopp et al., 2015)

148. Atlantal intercentrum, anteroventral lip: absent, anterior edge of intercentrum straight in lateral view (0); present, anterior edge of intercentrum concave (1).

(Wilson 2002, modified by Tschopp et al., 2015)

149. Atlantal intercentrum, ventral surface, foramen between posterior ventrolateral processes: absent (0); present (1).

(Tschopp et al., 2015)

150. Atlantal neurapophyses, anteromedial process: weakly developed (0); well-developed and distinct from posterior wing (1).

(Tschopp et al., 2015)

151. Atlantal neural arch, small subtriangular, laterally projecting spur at base: absent (0); present (1).

(Tschopp et al., 2015)

152. Atlantal neurapophyses, posterior wing: gradually tapering along its length (0); of subequal width along most of its length (1).

(Tschopp et al., 2015)

153. Atlantal neurapophyses, posterior wing: without foramen (0); with foramen (1).

(Wilson 2002; Whitlock 2011; wording modified by Tschopp et al., 2015)

154. Axial centrum, pneumatic fossae on ventrolateral edges, posterior and adjacent to parapophyses: absent (0); present (1).

(Tschopp et al., 2015)

155. Axis, prespinal lamina: of constant width (0); developing a transversely expanded, knob-like tuberosity at its anterior end (1).

(Tschopp et al., 2015)

156. Axis, postspinal lamina: absent (0); present (1).

(Harris & Dodson 2004)

157. Axis neural spine: projects beyond posterior border of centrum (0); terminates in front of or at posterior border of centrum (1); is restricted anterior to postzygapophyseal facets (2).

(Tschopp et al., 2015)

158. Anterior cervical vertebrae, total height/centrum length ratio: < 0.9 (0); 0.9-1.2 (1); > 1.2 (usually around 1.5) (2).

(Whitlock 2011, modified by Mannion et al. 2012)

159. Cervical vertebrae 2 and 3, centrum length: moderate length increase, CV3 < 1.3 x CV 2 (0); length increases considerably CV 3 at least 1.3 x CV 2 (1).

(Tschopp et al., 2015)

160. Anterior cervical vertebrae, posterior edge of anterior condyle: anteriorly inclined (0); posteriorly inclined (1).

(Tschopp et al., 2015)

161. Anterior cervical centra, pleurocoels: single (0); subdivided (1).

(Tschopp et al., 2015)

162. Anterior cervical vertebrae, pleurocoel extending onto dorsal surface of parapophysis: present (0); absent (1).

(Upchurch 1998, modified by Whitlock (2011), polarity reversed by Tschopp et al., 2015)

163. Anterior cervical vertebrae, longitudinal ridge on ventral surface: present (0); absent (1).

(Upchurch 1998, modified by Tschopp et al., 2015)

164. Anterior cervical vertebrae, paired pneumatic fossae on ventral surface: absent (0); present (1).

(Whitlock 2011)

165. Anterior cervical vertebrae, prespinal lamina: absent (0); present (1).

(Carballido et al. 2012)

166. Anterior and mid-cervical centra, pleurocoel pierced by one or two large, rounded foramina around centrum midlength: absent (0); present (1).

(Tschopp et al., 2015)

167. Anterior and mid-cervical vertebrae, spinoprezygapophyseal lamina, development at base of prezygapophyseal process: distinct (0); reduced to broad ridge or totally interrupted (1).

(Tschopp & Mateus (2013b: 103); wording modified by Tschopp et al., 2015)

168. Anterior and mid-cervical neural spines height: high (project well above the level of postzygapophyses) (0); low (terminates level with postzygapophyses) (1).

(Upchurch et al. 2004b, modified by Tschopp et al., 2015)

169. Anterior and mid-cervical neural spines, dorsoventrally elongate coel on lateral surface: absent (0); present (1).

(Mannion et al. 2012, modified by Tschopp et al., 2015)

170. Mid-cervical centra, anteroposterior length/height of posterior face: 2.5-3.2 (0); 3.3-4.4 (1); 4.5 + (2).

(Upchurch 1994, 1995, modified by Tschopp et al., 2015)

171. Mid-cervical pre-epipophyses anterior extreme: about the same as prezygapophyseal facet (0); projects considerably anterior to articular facet, forming a distinct spur (1).

(Sereno et al. 1999; Remes 2007; modified by Tschopp et al., 2015)

172. Mid-cervical neural spine height: considerably shorter than height of neural arch, <0.45 (0); subequal to height of neural arch, 0.45-1.6 (1); considerably higher than neural arch, >1.6 (2).

(Rauhut et al. 2005, modified by Whitlock 2011)

173. Mid-cervical neural spines, orientation: vertical (0); anteriorly inclined (1).

(Rauhut et al. 2005)

174. Mid-cervical vertebrae, angle between postzygodiapophyseal and spinopostzygapophyseal laminae: acute (0); right angle (1).

(Rauhut et al. 2005)

175. Mid- and posterior cervical centra, pleurocoel, subdivision: absent (0); present (1).

(Carballido et al., 2012 (C115), modified after Tschopp et al. 2015 (C171, fig. 36))

176. Mid- and posterior cervical centra, pleurocoel, subdivisions, if present: one, resulting in anterior and posterior excavations (0); many, resulting in complex morphology (1).

(Carballido et al., 2012 (C115), modified after Tschopp et al. 2015 (C171, fig. 36))

177. Mid- and posterior cervical vertebrae, pneumatization of lateral surface of centra: large, divided pleurocoel over approximately half of centrum (0); reduced, large fossa but sharp-bordered coel, if present, restricted to area above parapophysis (1).

(Whitlock 2011, modified by Tschopp et al., 2015)

178. Mid- and posterior cervical vertebrae, pleurocoel extending onto dorsal surface of parapophysis: present (0); absent (1).

(Upchurch 1998, modified by Mannion et al. (2012), based on Whitlock (2011))

179. Mid- and posterior cervical vertebrae, longitudinal ridge on ventral surface: present (0); absent (1).

(Tschopp et al., 2015)

180. Mid- and posterior cervical vertebrae, ventral keel: single (0); bifid, connects posterolaterally to the ventrolateral edges of the centrum (1).

(Tschopp et al., 2015)

181. Mid- and posterior cervical vertebrae, paired pneumatic fossae on ventral surface, separated by ventral midline keel: absent (0); present (1).

(Tschopp et al., 2015)

182. Mid- and posterior cervical vertebrae, lateral edge posterior to parapophysis: continuous (0); marked by a deep groove extending anteroposteriorly along the edge (1).

(Tschopp et al., 2015)

183. Mid- and posterior cervical centra, rugose tuberosity on anterodorsal corner of lateral side: absent (0); present (1).

(Tschopp & Mateus 2013; modified by Tschopp et al., 2015)

184. Mid- and posterior cervical centra with longitudinal flanges in the lateroventral edge on the posterior part of the centrum: absent (0); present (1).

(Tschopp & Mateus 2013)

185. Mid- and posterior cervical prezygapophyses, articular surfaces: flat (0); strongly convex transversely (1).

(Upchurch 1995, 1998)

186. Mid- and posterior cervical vertebrae, pre-epipophysis: absent (0); present (1).

(Remes 2007)

187. Mid- and posterior cervical vertebrae, spinoprezygapophyseal lamina, anterior end: remains vertical, with the free edge facing dorsally (0); is strongly inclined laterally (sometimes roofing a lateral fossa in the prezygapophyseal process (1).

(Tschopp & Mateus 2013, modified by Tschopp et al., 2015)

188. Mid- and posterior cervical neural arches, lateral fossae on the prezygapophysis process: absent (0); present (1).

(Harris 2006)

189. Mid- and posterior cervical vertebrae, prezygapophyseal centrodiapophyseal fossa, subdivision: absent, single cavity (0); present (1).

((Gilmore 1936); Upchurch et al. 2004b (C2); wording modified by Tschopp et al. 2015)

190. Mid- and posterior cervical vertebrae, prezygapophyseal centrodiapophyseal fossa, subdivision, if present: single accessory lamina, two subfossae (0); several accessory laminae, various smaller subfossae (1).

(Tschopp et al., 2015 (C184; figs 38, 40), modified)

191. Mid- and posterior cervical neural arches, centroprezygapophyseal lamina: single (0); dorsally bifurcated, with both divisions connected to prezygapophysis (1).

(Upchurch 1995, wording modified by Tscjopp et al. 2015)

192. Mid- and posterior cervical transverse processes: posterior centrodiapophyseal lamina (pcdl) and postzygodiapophyseal laminae (podl) meet at base of transverse process (0); pcdl and podl do not meet anteriorly, postzygapophyseal centrodiapophyseal fossa extends onto posterior face of transverse process (1).

(Tschopp et al., 2015)

193. Mid- and posterior cervical vertebrae, accessory horizontal lamina in center of spinodiapophyseal fossa, not connected with any surrounding laminae: absent (0); present (1).

(Tschopp et al., 2015)

194. Mid- and posterior cervical vertebrae, posterior centrodiapophyseal lamina: is single (0); bifurcates towards its anterior end (1).

(Upchurch et al. 2004b, modified by Tschopp et al., 2015)

195. Mid- and posterior cervical vertebrae, centropostzygapophyseal lamina (CPOL): single (0); divided, with the medial part contacting the interpostzygapophyseal lamina (1).

(Carballido et al., 2012)

196. Mid- and posterior cervical neural arches, interpostzygapophyseal lamina projects beyond the posterior margin of the neural arch (including the centropostzygapophyseal lamina), forming a prominent subrectangular projection in lateral view: absent (0); present (1).

(D'Emic 2012, modified by Mannion et al. 2013)

197. Mid- and posterior cervical vertebrae, postzygapophyseal centrodiapophyseal fossa and spinopostzygapophyseal fossa: entirely separated (0); connected by a large foramen (1).

(Tschopp et al., 2015)

198. Posterior cervical vertebrae, Elongation Index (cervical centrum length, excluding condyle, divided by posterior centrum height): less than 2.0 (0); 2.0 - 2.6 (1); higher than 2.6 (2).

(Gauthier 1986; Mannion et al. 2012, modified by Tschopp et al., 2015)

199. Posterior cervical vertebrae, ventral keel: anteriorly placed (0); restricted to posterior portion of centrum (1).

(Tschopp et al., 2015)

200. Posterior cervical prezygapophyses: terminate with or in front of articular ball of centrum (0); terminate well behind articular ball (1).

(Upchurch et al. 2004b)

201. Posterior cervical vertebrae, prezygapophysis articular facet posterior margin: confluent with prezygapophyseal process (0); bordered posteriorly by conspicuous transverse sulcus (1).

(Tschopp & Mateus 2013)

202. Posterior cervical vertebrae, spinoprezygapophyseal lamina: continuous (0); developing an anterior projection (just beneath but independent from the spine summit) (1).

(Tschopp & Mateus 2013)

203. Posterior cervical vertebrae, accessory lateral lamina connecting postzygodiapophyseal and spinoprezygapophyseal laminae: absent (0); present (1).

(Gallina & Apesteguía 2005)

204. Posterior cervical vertebrae, accessory, subvertical lamina in the postzygapophyseal centrodiapophyseal fossa, with free edge facing laterally: absent (0); present (1).

(Mannion et al. 2012)

205. Posterior cervical vertebrae, accessory, subvertical lamina in the postzygapophyseal centrodiapophyseal fossa, with free edge facing posteriorly: absent (0); present (1).

(Gilmore 1936; Upchurch et al. 2004b; modified by Tschopp et al., 2015)

206. Posterior cervical postzygapophyses: terminate at or beyond posterior edge of centrum (0); terminate in front of posterior edge (1).

(Upchurch et al. 2004b, modified by Tschopp & Mateus 2013)

207. Posterior cervical neural arch, interpostzygapophyseal lamina (tpol): connects directly with roof of neural canal (0); vertical lamina connects tpol with neural canal roof (1).

(Tschopp et al., 2015)

208. Posterior cervical neural arches, epipophyses: transversely compressed (0); dorsoventrally compressed (1).

(Tschopp et al., 2015)

209. Posterior cervical neural arches, accessory spinal lamina: absent (0); present, running vertically just posterior to spinoprezygapophyseal lamina (1).

(Whitlock 2011)

210. Posterior cervical neural spines, dorsoventrally elongate coel on lateral surface: absent (0); present (1).

(Mannion et al. 2012)

211. Posterior cervical neural spines, horizontal, rugose ridge right below spine summit on lateral surface: absent (0); present, serves as distinct dorsal edge of the spinodiapophyseal fossa (1).

(Tschopp & Mateus 2013)

212. Posterior bifid, cervical neural spines, medial surface: marked by distinct, dorsoventral ridge from base to spine summit (0); smooth (1).

(Tschopp et al., 2015)

213. Posterior cervical neural and/or anterior-most dorsal neural spines: vertical (0); anteriorly inclined (1).

(Rauhut et al. 2005)

214. Posterior cervical and anterior dorsal vertebrae, roughened lateral aspect of prezygodiapophyseal lamina: absent (0); present (1).

(Whitlock 2011)

215. Posterior cervical and anterior dorsal vertebrae, prespinal lamina: absent (0), present (1).

(Salgado et al. 1997)

216. Posterior cervical and anterior dorsal bifid neural spines, median tubercle: absent (0); present (1).

(McIntosh 1990a; Upchurch 1995)

217. Posterior cervical and anterior dorsal bifid neural spines, orientation: diverging (0); parallel to converging (1).

(Rauhut et al. 2005, modified by Tschopp et al., 2015)

218. Posterior cervical and anterior dorsal bifid neural spines, divergence: wide (0); narrow, distance between spine summits subequal to neural canal width (1).

(Rauhut et al. 2005, modified by Tschopp et al., 2015)

219. Posterior cervical, and anterior and mid-dorsal vertebrae, anterior projection of diapophysis laterally adjacent to prezygapophyseal facet: absent (0); present (1).

(Tschopp et al., 2015)

220. Cervical ribs, length: long, reaching posterior to posterior end of centrum (0); short, not reaching posterior end of centrum (1).

(Russell & Zheng 1993, modified by Tschopp et al., 2015)

221. Cervical ribs, length: overlapping several centra posterior (0); overlapping no more than the next cervical vertebra in sequence (1).

(Russell & Zheng, 1993, modified by Tschopp et al., 2015)

222. Cervical ribs, position relative to centrum: not projecting far beneath centrum (0); projecting well beneath centrum, such that length of posterior process is subequal in length to fused diapophysis/tuberculum (1).

(Wilson 2002, Whitlock 2011)

223. Cervical ribs, posteriorly projecting spur on dorsolateral edge of posterior shaft: absent (0); present (1).

(Tschopp et al., 2015)

224. Anterior and mid-cervical ribs, tuberculum in lateral view: is directed nearly vertically (0); is directed upwards and backwards (1).

(Upchurch et al. 2004b, modified by Tschopp et al., 2015)

225. Posterior cervical ribs, anterior process: present (0); absent (1).

(Upchurch et al. 2004b (C9), wording modified by Tschopp et al., 2015 (C219))

226. Posterior cervical ribs, anterior process: distinct, much longer anteroposteriorly than high dorsoventrally (0); reduced to a short bump-like process or absent (1).

(Tschopp et al., 2015)

227. Posterior cervical ribs, anterior process: rounded in lateral view (0); has an acute pointed tip in lateral view (1).

(Upchurch et al. 2004b (C10), wording modified by Tschopp et al., 2015 (C221))

228. Posterior cervical ribs, rounded sub-triangular process in lateral view, posteroventral to tuberculum: absent (0); present (1).

(Wedel & Sanders 2002; Upchurch et al. 2004b, wording modified by Tschopp et al., 2015)

229. Posterior cervical rib shafts: nearly straight and directed backward and a little upwards (0); initially directed in same direction but turn to run a little downwards toward distal tip (1).

(Upchurch et al. 2004b)

230. Number of dorsal vertebrae: 13 or more (0); 12 (1); 10 (2); 9 (3).

(McIntosh 1990a; Russell & Zheng 1993, modified by Upchurch 1998, Wilson & Sereno 1998)

231. Dorsal centrum length (excluding articular 'ball'), remains approximately the same along the sequence (0); shortens from anterior to posterior dorsal vertebrae (1).

(Mannion et al. 2012)

232. Dorsal vertebrae, opisthocoely (including a prominent anterior articular 'ball') disappears: between DV2 and DV3 (0); between DV3 and DV4 or more posteriorly (1).

(Holland 1915a, Gilmore 1936, Upchurch et al. 2004b)

233. Dorsal pneumatopores (pleurocoels): absent (0); present (1).

(Gauthier 1986; McIntosh 1990a; Upchurch 1995)

234. Dorsal centra, internal pneumaticity: absent (0); present (1).

((Cope 1877); Wilson 2002 (C77), modified after Carballido et al. 2012 (C139))

235. Dorsal centra, internal pneumaticity, structure: simple, large cavities (0); complex, many small cavities (1).

((Cope 1877); Wilson 2002 (C77), modified after Carballido et al. 2012 (C139))

236. Dorsal neural arches, paired, subdivided pneumatic chambers dorsolateral to neural canal: absent (0), present (1).

(Sereno et al. 1999; Whitlock 2011)

237. Dorsal transverse processes, orientation: horizontal or only slightly inclined dorsally (0); more than 30° inclined dorsally from the horizontal (1).

(Yu 1993, modified by Upchurch 1998)

238. Dorsal vertebrae, single (not bifid) neural spines, spinoprezygapophyseal laminae: separate along entire length (0); joined distally, forming single prespinal lamina (1).

(Upchurch 1995, modified by Whitlock 2011)

239. Dorsal vertebrae, spinodiapophyseal webbing: laminae follow curvature of neural spine and diapophysis in anterior view (0); laminae 'festooned' from spine, dorsal margin does not closely follow shape of neural spine and diapophysis (1).

(Sereno et al. 2007)

240. Dorsal vertebrae with single neural spines in anterior view, middle single fossa projected through midline of neural spine: present (0); absent (1).

(Carballido et al., 2012)

241. Dorsal (single) neural spines, postspinal lamina, dorsal end: flat to convex transversely (0); concave transversely (1).

(Tschopp et al., 2015)

242. Dorsal vertebrae, transition from bifid to single neural spines: gradual (0); abrupt (1).

(Tschopp et al., 2015)

243. Dorsal neural arches, hyposphene-hypantrum articulations: present (0); absent (1).

(Gauthier 1986; Salgado et al. 1997)

244. Dorsal vertebrae, hyposphene first appears: on D3 (0); on D4 or more posteriorly (1).

(Upchurch et al. 2004b, modified by Tschopp et al., 2015)

245. Dorsal vertebrae, single vertical lamina supporting the hyposphene from below: absent (0); present (1).

(Gilmore 1936, Upchurch et al. 2004b, modified by Tschopp et al., 2015)

246. Dorsal vertebrae 1 and 2, centrum length: DV 1 > DV 2 (0); DV 2 > DV 1 (1).

(Gilmore 1936; Upchurch et al. 2004b)

247. First dorsal vertebrae, pleurocoel location: occupy the anterior and middle part of the centrum (0); occupy the posterior part of the centrum (1).

(Holland 1915a, Gilmore 1936, Upchurch et al. 2004b (C17), wording modified by Tschopp et al., 2015 (C240))

248. Anterior dorsal vertebrae, pleurocoels in first few centra: become larger along the series (0); become smaller (1).

(Gilmore 1936, Upchurch et al. 2004b, modified by Tschopp et al., 2015)

249. Anterior dorsal vertebrae, ventral keel: absent (0); present (1).

(Mannion et al. 2012)

250. Anterior dorsal transverse process position: high, considerably above dorsal edge of posterior cotyle (0); low, ventral edge about level to dorsal edge of posterior cotyle (1).

(Gilmore 1936)

251. Anterior, bifid dorsal vertebrae, base of notch between metapophyses: wide and rounded (0); narrow, V-shaped (1).

(Gilmore 1936)

252. Anterior dorsal, bifid neural spines, medial surface: gently rounded transversely (0); subtriangular (1).

(Tschopp et al., 2015)

253. Dorsal vertebra 3, parapophysis: lies at the top of the centrum (0); lies mid-way between the top of the centrum and the level of the prezygapophyses (1).

(Gilmore 1936, Upchurch et al. 2004b (C18), wording modified by Tschopp et al., 2015 (C246))

254. Anterior and mid-dorsal centra, pleurocoels: situated entirely on centrum (0); invade neural arch pedicels (1).

((Holland 1915); Tschopp et al. 2015)

255. Anterior and mid-dorsal neural arch, hyposphene shape: rhomboid (0); laminar (1).

(Tschopp et al., 2015)

256. Mid-dorsal neural arches, height above postzygapophyses (neural spine) to height below (pedicel): 2.1 or greater (0); < 2.1 (1).

(Whitlock 2011)

257. Mid-dorsal neural spines, form: single, bifid form (if present) does not extend past second or third dorsal (0); bifid, inclusive of at least fifth dorsal vertebrae (1).

(Whitlock 2011)

258. Mid-dorsal neural spines, oblique accessory lamina connecting postspinal lamina with spinopostzygapophyseal lamina: absent (0); present (1).

(Tschopp et al., 2015)

259. Mid- and posterior dorsal vertebrae, lateral pleurocoels present in centra: absent (0); present (1).

(Gauthier 1986; McIntosh 1990a; Upchurch 1995, modified by Whitlock 2011)

260. Mid- and posterior dorsal vertebrae, vertically oriented rod-like struts divide the lateral pneumatic foramina: absent (0); present (1).

(Mannion et al. 2012)

261. Mid- and posterior dorsal vertebrae, height of neural arch below postzygapophyses (pedicel)/posterior cotyle height: <0.8 (0); 0.8 or greater (1).

(Gallina & Apesteguía 2005, modified by Tschopp et al., 2015)

262. Mid- and posterior dorsal neural arches, prezygoparapophyseal lamina: present (0); absent (1).

(Wilson 2002)

263. Mid- and posterior dorsal parapophyses, location: above centrum, posterior to anterior edge of centrum (0); straight above anterior edge of centrum, or anteriorly displaced (1).

(Tschopp et al., 2015)

264. Mid- and posterior dorsal neural arches, anterior centroparapophyseal lamina: absent (0); present (1).

(Upchurch et al. 2004a; Mannion et al. 2013)

265. Mid- and posterior dorsal neural arches, posterior centroparapophyseal lamina: absent (0); present (1).

(Salgado et al. 1997, modified by Mannion et al. 2013, modified (split))

266. Mid- and posterior dorsal neural arches, posterior centroparapophyseal lamina, if present: single (0); double (1)

(Salgado et al. 1997, modified after Mannion et al. 2013 by Tschopp et al. 2015, modified (split))

267. Mid- and posterior dorsal vertebrae, accessory laminae in region between posterior centrodiapophyseal lamina and posterior centroparapophyseal lamina: absent (0); present (1).

(Mannion et al. 2012)

268. Mid- and posterior dorsal vertebrae, accessory lamina linking hyposphene with base of posterior centrodiapophyseal lamina: absent (0); present (1).

(Tschopp et al., 2015)

269. Mid- and posterior dorsal neural arches, centropostzygapophyseal lamina: single (0); divided, lateral branch connecting to posterior centrodiapophyseal lamina (1).

(Wilson 2002, wording modified by Tschopp et al., 2015)

270. Mid- and posterior dorsal neural arches, infradiapophyseal pneumatopore between anterior and posterior centrodiapophyseal laminae: absent (0); present (1).

(Wilson 2002)

271. Mid- and posterior dorsal transverse processes, length: short (0); long (projecting > 1.3 times posterior cotyle width) (1).

(Carballido et al., 2012, modified by Tschopp et al., 2015)

272. Mid- and posterior dorsal transverse processes, dorsal edge: straight, or curving downwards at distal end (0); developing a distinct dorsal bump or spur (1).

(Tschopp et al., 2015)

273. Mid- and posterior dorsal neural spines, anteroposterior width: approximately constant along height of spine, with subparallel anterior and posterior margins (0); narrows dorsally to form triangular shape in lateral view, with base being approximately twice the width of dorsal tip (1).

(Mannion et al., 2013, based on Taylor 2009; modified by Tschopp et al., 2015)

274. Mid- and posterior dorsal neural spines, accessory, anterodorsally oriented lamina arising from the SPDL: absent (0); present (1).

((Hanik et al. in review))

275. Mid- and posterior dorsal neural spines, breadth at summit: much narrower (0); equal to or broader (1) transversely than anteroposteriorly.

(Wilson 2002, modified by Tschopp et al., 2015)

276. Mid- and posterior dorsal neural spines, triangular aliform processes: absent (0); present (1).

(Upchurch 1998, modified after Carballido et al. (2012))

277. Mid- and posterior dorsal neural spines, triangular aliform processes, lateral extension: do not project as far laterally as postzygapophyses (0); project at least as far laterally as postzygapophyses (1).

(Upchurch 1998, modified by Tschopp et al. 2015, after Carballido et al. (2012))

278. Posterior dorsal centra, total length/height of posterior articular surface: 1.0 or greater (0); short, < 1.0 (1).

(Tschopp et al., 2015)

279. Posterior dorsal centra, posterior articular surface width to height: 1.0 or less (0); >1.0 (1).

((Gilmore 1936); Tschopp et al. 2015)

280. Posterior dorsal centra, articular face shape: amphicoelous (0); slightly opisthocoelous (1); strongly opisthocoelous (2).

(Yu 1993, wording modified by Carballido et al. 2012)

281. Posterior dorsal vertebrae, pleurocoel shape: oval to circular (0); subtriangular with apex dorsally (1).

(Tschopp et al., 2015)

282. Posterior dorsal neural arches, height above postzygapophyses (neural spine) to height below (pedicel): < 3.1 (0); 3.1 or greater (1).

(Tschopp et al., 2015)

283. Posterior dorsal neural arches, parapophyseal centrodiapophyseal fossa: ventrally open, relatively shallow (0); deep, triangular (1).

(Gallina & Apesteguía 2005)

284. Posterior dorsal vertebrae, spinoprezygapophyseal lamina: absent or greatly reduced (0); present (1).

(Upchurch et al. 2007, modified by Tschopp et al., 2015)

285. Posterior dorsal postzygapophyses: almost horizontal, such that the two articular facets include a wide angle (0); articular facets oblique, including an almost 90° angle (1).

(Tschopp et al., 2015)

286. Posterior dorsal vertebrae, hyposphene-hypantrum system: well developed, rhomboid shape up to last element (0); weakly developed, mainly as a laminar articulation (1).

(Carballido et al., 2012, modified by Tschopp et al., 2015)

287. Posterior dorsal neural arches, spinopostzygapophyseal laminae: single (0); divided near postzygapophyses (1).

(Wilson 2002)

288. Posterior dorsal vertebrae, medial spinopostzygapophyseal lamina: absent (0); present and forms part of median posterior lamina (1).

(Carballido et al. 2012)

289. Posterior dorsal vertebrae, base of neural spines just above transverse processes: longer than wide (0); subequal in width and length (1).

(Tschopp et al., 2015)

290. Posterior dorsal neural spines, orientation at its base: vertical (0); anteriorly inclined (1).

(Tschopp et al., 2015)

291. Posterior dorsal neural spines, midline cleft along the dorsal surface: absent (0); present (1).

(Mannion et al. 2012)

292. Posterior dorsal and/or sacral neural spines (not including arch), height: less than 2 times centrum length (0); 2 to 3 times centrum length (1); more than 3 times centrum length (2).

(Mannion et al. 2012, modified by Tschopp et al., 2015)

293. Dorsal ribs, rib head: area between capitulum and tuberculum flat (0); oblique ridge present that connects medial and lateral edge at the base of the rib head (1).

(Tschopp et al., 2015)

294. Dorsal ribs, proximal pneumatopores: absent (0); present (1).

(Wilson 2002)

295. Mid-dorsal ribs, orientation of tuberculum: spreading outside from rib shaft (0); following straight direction of rib shaft (1); following medial bend of rib shaft (2).

(Gallina & Apesteguía 2005)

296. Sacral vertebrae, number: 4 (0); 5 (1); 6 (2).

(Salgado et al. 1997, modified by Tschopp et al., 2015)

297. Sacral vertebral centra, pleurocoels: absent (0); present (1).

(Upchurch et al., 2004a, wording modified by Tschopp et al., 2015)

298. Sacral rib III, ventral surface: smooth (0); with oblique ridge (1).

(Mook 1917; Tschopp et al. 2015)

299. Sacral neural spines, lateral side, towards summit: flat, with only spinodiapophyseal lamina (spdl) well-developed (0); with distinct horizontal accessory laminae that connect spdl to pre- and/or postspinal lamina (1).

(Tschopp et al., 2015)

300. Sacral neural spines, lateral view, spinodiapophyseal lamina: reduced to absent, does not connect summit and diapophysis (0); present and distinct, connects spine summit with diapophysis (1).

(Tschopp et al., 2015)

301. Sacral neural spines, lateral view, spinodiapophyseal laminae (spdl): remain vertical and thus parallel to each other (0); spdl of neighboring spines converge (1).

(Tschopp et al., 2015)

302. Caudal neural spines, elliptical depression between lateral spinal lamina and postspinal lamina on dorsolateral surface: absent (0); present (1).

(Sereno et al. 2007)

303. Caudal neural spines with triangular lateral processes: absent (0); present (1).

(Sereno et al. 2007)

304. Posterior dorsal, sacral and anterior caudal neural spines, shape in anterior/posterior view: rectangular through most of length (0); 'petal' shaped, expanding transversely through 75% of its length and then tapering (1).

(Calvo & Salgado 1995; Upchurch 1998)

305. First caudal centrum, articular face shape: procoelous (0); flat (1); opisthocoelous (2).

(Salgado et al. 1997)

306. Anterior-most caudal centra, transverse cross-section: sub-circular with rounded ventral margin (0); 'heart'-shaped with an acute ventral ridge (1).

(Gilmore 1936, Upchurch et al. 2004b (C22), modified by Tschopp et al., 2015 (C296))

307. Anterior-most caudal centra, pneumatic fossae: reduced to absent (0); large pleurocoels (1).

(Tschopp et al., 2015)

308. Anterior-most caudal vertebrae, additional pneumatic fossa on posterodorsal corner of centrum: absent (0); present (1).

(Tschopp et al., 2015)

309. Anterior-most caudal transverse processes, shape: triangular, tapering distally (0); wing-like (1).

(McIntosh 1990a, Yu 1993, modified by Tschopp et al., 2015)

310. Anterior-most caudal vertebrae, transition from 'fan'-shaped to ' normal' caudal ribs: between Cd 1 and 2 (0); Cd4 and Cd5 (1); Cd5 and Cd6 (2); Cd6 and Cd7 (3); Cd7 and Cd8 or more posteriorly (4).

(Upchurch et al. 2004b, modified by Tschopp et al., 2015)

311. Anterior-most caudal neural arches, accessory lamina connecting pre- and postzygapophyses: absent (0); present (1).

(Tschopp et al., 2015)

312. Anterior-most caudal neural spine (not including arch), height: less than 1.5 times centrum height (0); 1.5 times centrum height or more (1).

(Yu 1993, modified by Tschopp et al., 2015, after Upchurch & Mannion 2009)

313. Anterior-most caudal neural spines, lateral spinal lamina: has the same anteroposterior width ventrally and dorsally (0); expands anteroposteriorly towards its distal end, and becomes rugose (1).

(Upchurch et al. 2004b)

314. Anterior caudal centra (excluding the first), articular surface shape: amphiplatyan or amphicoelous (0); procoelous/distoplatyan (1); slightly procoelous (2); procoelous (3).

(McIntosh 1990a; Russell & Zheng 1993, modified by Carballido et al. 2012, after Gonzáles Riga et al. 2009)

315. Anterior caudal centra, ventral surface: without irregularly placed foramina (0); irregular foramina present on some anterior caudal vertebrae (1).

(Whitlock 2011)

316. Anterior caudal centra, pneumatopores (pleurocoels): absent (0); present (1).

(McIntosh 1990a, Yu 1993, modified by Tschopp et al., 2015)

317. Anterior caudal centra, pneumatopores: restricted to foramina (0); large coels present (1).

(Tschopp & Mateus 2013)

318. Anterior caudal centra, pneumatopores: disappear by caudal 15 (0); present until caudal 16 or more (1).

(McIntosh 2005)

319. Anterior caudal centra, length: subequal amongst first 20 (0); more or less doubling over first 20 (1).

(Upchurch 1998)

320. Anterior caudal vertebrae, concavo-convex zygapophyseal articulation: absent (0); present (1).

(Wilson 2002; Whitlock 2011)

321. Anterior caudal prezygapophyses, pre-epipophysis laterally below articular facet: absent (0); present (1).

(Tschopp et al., 2015)

322. Anterior caudal vertebrae, transverse processes: ventral surface directed laterally or slightly ventrally (0); directed dorsally (1).

(Whitlock 2011)

323. Anterior caudal transverse processes, anterior diapophyseal laminae (acdl, prdl): reduced or absent (0); present, well defined (1).

(Wilson 2002)

324. Anterior caudal transverse processes, anterior centrodiapophyseal lamina, shape: single (0); divided (1).

(Wilson 2002)

325. Anterior caudal transverse processes, posterior diapophyseal laminae (pcdl, podl): reduced or absent (0); present, well defined (1).

(Whitlock 2011, modified by Tschopp et al., 2015)

326. Anterior caudal transverse processes, anteroposteriorly expanded lateral extremities: absent (0); present (1).

(Tschopp et al., 2015)

327. Anterior caudal neural spines, maximum mediolateral width to anteroposterior length ratio: < 1.0 (0); 1.0 or greater (1).

(Upchurch 1998, modified by Mannion et al. 2013)

328. Anterior caudal neural spines, spinoprezygapophyseal lamina: absent, or present as small short ridges that rapidly fade out into the anterolateral margin of the spine (0); present, extending onto lateral aspect of neural spine (1).

(Wilson 2002, modified by Mannion et al. 2012)

329. Anterior caudal neural spines, spinopre- and spinopostzygapophyseal laminae contact: absent (0); present (1).

(Wilson 2002)

330. Anterior caudal neural arches, prespinal lamina: absent (0); present (1).

(Upchurch 1995)

331. Anterior caudal neural spines, thickened anterior rim of prespinal lamina: absent (0); present (1).

(Gallina & Apesteguía 2005)

332. Anterior caudal neural spines, prespinal lamina or rugosity: terminate at or beneath dorsal margin of neural spine (0); project dorsally above neural spine (1).

(Whitlock 2011, modified by Tschopp et al., 2015)

333. Anterior caudal neural arches, postspinal lamina: absent (0); present (1).

(Upchurch 1995)

334. Anterior caudal neural spines, postspinal lamina or rugosity: terminate at or beneath dorsal margin of neural spine (0); project dorsally above neural spine (1).

(Whitlock 2011, modified by Tschopp et al., 2015)

335. Anterior caudal neural arches; hyposphenal ridge on posterior face of neural arch; present (0); absent (1).

(Mannion et al. 2012)

336. Anterior caudal neural spines, shape: single (0); slightly bifurcate anteriorly (1).

(Whitlock 2011)

337. Anterior caudal neural spines, maximum mediolateral width to minimum mediolateral width ratio: < 2.0 (0); 2.0 or greater (1).

(Canudo et al. 2008; Taylor 2009, modified by Mannion et al., 2013)

338. Anterior caudal neural spines, lateral expansion at distal end: gradual, expanding through the last third of the neural spine (0); abrupt, restricted to distal fourth of neural spine (1).

(Tschopp et al., 2015)

339. Anterior and mid-caudal vertebrae, ventrolateral ridges: absent (0); present (1).

(Upchurch et al. 2004a)

340. Anterior and mid-caudal centra, ventral longitudinal hollow: absent (0); present (1).

(McIntosh 1990a, Yu 1993)

341. Anterior- and mid-caudal vertebrae, ventral hollow depth: shallow, 10mm or less (0); deep, >10mm (1).

(Curtice 1996; Tschopp et al. 2015)

342. Mid-caudal vertebrae, ratio of centrum length to posterior height: < 1,7 (0); 1,7 or greater (1).

(Yu 1993, modified by Upchurch & Martin 2003)

343. Mid-caudal vertebrae, lateral surface of centra: without longitudinal ridge at midheight (0); longitudinal ridge present, centra hexagonal in anterior/posterior view (1).

(Upchurch & Martin 2002)

344. Mid-caudal centra, articular surface shape: cylindrical (0); quadrangular (1); trapezoidal (2); with flat ventral margin but rounded lateral edges (3).

(Wilson 2002; Gallina & Apesteguía 2005; modified by Tschopp et al., 2015, after Carballido et al. (2012))

345. Mid-caudal centra ventral surface in lateral view: gently curved (0); greater portion straight, with expansions on both ends to form the chevron facets restricted to about last fourth of centrum length (1).

(Tschopp et al., 2015)

346. Mid-caudal posterior articular surface: concave (0); flat (1); convex (2).

(Tschopp et al., 2015)

347. Mid-caudal neural arches: over the midpoint of the centrum with approximately subequal amounts of the centrum exposed at either end (0); on the anterior half of the centrum (1).

(Huene 1929; Salgado et al. 1997)

348. Mid-caudal prezygapophyses: free (0); posteriorly interconnected by a transverse ridge, creating a triangular fossa together with the spinoprezygapophyseal laminae (1).

(Tschopp et al., 2015)

349. Mid-caudal prezygapophyses position: terminate at or behind anterior edge of centrum (0); project considerably beyond anterior edge of centrum (1).

(Tschopp et al., 2015)

350. Mid-caudal neural spines, orientation: directed posteriorly (0); vertical (1).

(McIntosh 1990b; Salgado et al. 1997, modified by Tschopp et al., 2015, after Carballido et al. (2012))

351. Mid-caudal neural arch, anterior extreme of spine summit: smooth (0); developing a short anterior or anterodorsal projection, such that anterior edge of spine becomes slightly concave (1).

(Tschopp et al., 2015)

352. Mid- and posterior caudal vertebral centra, articular surfaces: subequal in width and height or higher than wide (0); considerably wider than high (1).

(Salgado et al. 1997, modified by Tschopp et al., 2015)

353. Mid- and posterior caudal neural spines: spine summit overhangs postzygapophyses considerably posteriorly (0); posterior end of spine summit more or less straight above postzygapophyses (1).

(Tschopp et al., 2015)

354. Mid- and posterior caudal spines: elongate and strongly caudally directed, extending over more than 50% of length of succeeding vertebral centrum (0); short, not extending far beyond caudal articular facet of centrum (1).

(Remes et al. 2009, polarity inverted by Tschopp et al., 2015)

355. Posterior caudal prezygapophyses position: terminate at or behind anterior edge of centrum (0); project beyond anterior edge of centrum (1).

(Tschopp et al., 2015)

356. Distal-most caudal centra, articular face shape: platycoelous (0); biconvex (1).

(Wilson et al. 1999)

357. Distal-most caudal centra, length-to-height ratio: < 4.0 (0); 4.0-6.5 (1); > 6.5 (2).

(Upchurch 1998, modified by Tschopp et al., 2015, after Wilson et al., 1999)

358. Distal-most biconvex caudal centra, number: ten or fewer (0); more than 30 (1).

(Wilson 2002)

359. Caudal ribs, last occurs on: Cd 12 or more anteriorly (0); Cd 13 (1); Cd 14 (2); Cd 15-17 (3); Cd 18 or more posteriorly (4).

(Holland 1915a, Gilmore 1936, Upchurch et al. 2004b)

360. Anterior, 'fan'-shaped caudal ribs, foramen: absent (0); present (1).

(Gilmore 1936, Upchurch et al. 2004b (C25); polarity reversed by Tschopp et al. 2015 (C350))

361. Chevrons, 'crus' bridging haemal canal: absent in some (0); present in all (1).

(Yu 1993, modified by Tschopp et al., 2015)

362. Chevrons, 'crus' bridging haemal canal: present in some (0); absent in all (1).

(Yu 1993, modified by Tschopp et al., 2015)

363. Chevrons with anterior and posterior projections: present (0); absent (1).

(McIntosh 1989; Russell & Zheng 1993, modified by Wilson 2002)

364. Anterior chevrons, longitudinal median ridge on anterior surface: absent (0); present (1).

(Tschopp et al., 2015)

365. Anterior chevrons, posterior edge of distal blade in lateral view: continuous (0); posteriorly expanded in a step-like fashion (1).

(Tschopp et al., 2015)

366. Anterior mid-chevrons, lateral surface: smooth (0); marked by a horizontal ridge right below articulation surfaces (1).

(Tschopp et al., 2015)

367. Middle chevrons, distinct fossae on medial surfaces of proximal branches: absent (0); present (1).

(Tschopp et al., 2015)

368. Forked chevrons, anteroposterior length: short, about 50% of relative vertebral centrum length (0); elongate, approaching corresponding vertebral centrum length (1).

(McIntosh 1995)

369. Scapular length/minimum blade breadth: > 5.5 (0); 5.5 or less (1).

(Carballido et al., 2012)

370. Scapular acromion length/scapular length: > 0.54 (0); 0.46-0.54 (1); < 0.46 (2).

(Gallina & Apesteguía 2005, modified by Tschopp et al., 2015)

371. Scapula, orientation of blade, angle with coracoid articulation: > 80° (0); 80° or less (1).

(Wilson 2002, modified by Tschopp et al., 2015)

372. Scapula, angle between acromial ridge and distal blade: < 70° (0); 70°-81° (1); > 81° (2).

(Riggs 1903, Carpenter & McIntosh 1994, Upchurch et al. 2004b)

373. Scapular acromion process, dorsal part of posterior margin: convex or straight (0); U-shaped concavity (1).

(Wilson 2002, modified by Whitlock 2011)

374. Scapular, acromion process position: lies near glenoid level (0); lies nearly at midpoint of scapular body (1).

(Carballido et al., 2012)

375. Scapula, area posterior to acromial ridge and distal blade: is excavated (0); is flat or slightly convex (1).

(Upchurch et al. 2004b)

376. Scapular glenoid, orientation: relatively flat or laterally facing (0); strongly beveled medially (1).

(Wilson & Sereno 1998)

377. Scapular blade, acromial edge, shape in lateral view: straight (0); concave (1).

(Wilson 2002 (C152), wording modified by Tschopp et al. 2015 (C367; Fig. 87), split in three characters)

378. Scapular blade, acromial edge, distinct dorsal process close to distal end: absent (0); present (1).

(Wilson 2002 (C152), wording modified by Tschopp et al. 2015 (C367; Fig. 87), split in three characters)

379. Scapular blade, ventral edge, shape in lateral view: is straight (0); curves ventrally towards its distal end (1).

(Upchurch et al. 2004b; wording modified)

380. Scapula: without semi-ovate, flat muscle scar just distal to glenoid on scapular shaft (0); scar present (1).

(Whitlock 2011)

381. Scapular blade, subtriangular projection on anterior portion of ventral edge: absent (0); present (1).

(Gallina & Apesteguía 2005)

382. Scapular blade, expansion of distal end: wide (at least 2 times narrowest width of shaft in lateral view) (0); narrow (< 2 times narrowest width of shaft) (1).

(Yu 1993, modified by Upchurch et al. 2004b)

383. Scapular blade, distal end, shape: with distinct dorsal and ventral corners (0); widely rounded (1).

(Wilson 2002 (C152), wording modified by Tschopp et al. 2015 (C367; Fig. 87), split in three characters)

384. Coracoid, anteroventral margin shape: rounded (0); rectangular (1).

(Salgado et al. 1997)

385. Coracoid, infraglenoid deep groove: reduced to absent (0); present and distinct (1).

(Carballido et al., 2012, modified by Tschopp et al., 2015)

386. Sternal plates, shape: subcircular or oval (0); subtriangular with widened posterior border (1); elliptical to crescentic, with concave lateral margin (2).

(Calvo & Salgado 1995, modified by Tschopp et al., 2015)

387. Sternal plate, ridge on the ventral surface: absent (0); broad and shallow, or elongate and prominent (1).

(Upchurch et al., 2004a, wording modified by Tschopp et al., 2015)

388. Sternal plate, anterior end: expanded dorsoventrally (0); flat, not expanded (1).

(Tschopp & Mateus 2012, modified by Tschopp et al., 2015)

389. Sternal plate, posterior border: convex (0); straight (1).

(Gonzáles-Riga 2002, modified by Tschopp et al., 2015)

390. Forelimb: hindlimb length ratio: 0.76 or greater (0); less than 0.76 (1).

(Upchurch 1995, 1998)

391. Humerus-to-femur ratio: < 0.7 (0); 0.7-0.76 (1); 0.77-0.89 (2); = or > 0.90 (3).

(McIntosh 1990, modified by Tschopp et al., 2015)

392. Humerus, RI (sensu Wilson and Upchurch, 2003): Gracile (less than 0,27) (0); medium (0,28-0,32) (1); Robust (more than 0,33) (2).

(Carballido et al., 2012)

393. Humerus, shaft twist: minor to absent (0); high, distal articular surface twisted by at least 40° compared to proximal articular surface (1).

(Gilmore, 1932; Tschopp et al. 2015)

394. Humerus, midshaft cross-section, shape: circular, transverse diameter: anteroposterior diameter ratio is 1.5 or lower (usually close to 1.3) (0); elliptical, transverse diameter: anteroposterior diameter ratio is greater than 1.5 (usually close to 1.8) (1).

(Wilson 2002, modified by Mannion et al. 2012)

395. Humerus, pronounced proximolateral corner: absent (0); present (1).

(Upchurch 1998)

396. Humerus, proximal expansion: more or less symmetrical (0); asymmetrical, proximomedial corner much more pronounced than proximolateral one (1).

(Wilhite 2005; Tschopp et al. 2015)

397. Humerus, proximal end expanded laterally in anterior/proximal view: expanded, lateral margin concave in anterior/posterior view (0); not expanded (1).

(Curry Rogers 2005)

398. Humerus, shallow, but distinct rugose tubercle at the center of the concave proximal portion of the anterior surface: absent (0); present (1).

(Tschopp et al., 2015)

399. Ulna to humerus length: < 0.65 (0); 0.66-0.76 (1); > 0.76 (2).

(Janensch 1929; Tschopp et al. 2015)

400. Ulna, proximal condylar processes: subequal in length (0); anterior arm longer (1).

(Wilson 2002)

401. Ulna, proximal articular surface, angle between anterior and lateral branch: 90° (0); acute (1).

(Tschopp et al., 2015)

402. Ulna, distal transverse expansion: slight, < 1.3 times min sw (0); wide, 1,3 times min sw or greater (1).

(Tschopp et al., 2015)

403. Radius, maximum diameter of the proximal end divided by greatest length: < 0.3 (0); 0.3 or greater (1).

((McIntosh 1990a), Mannion et al. 2013)

404. Radius, distal articular surface for ulna: reduced and relatively smooth (0); well developed with one or two distinct longitudinal ridges (1).

(Tschopp et al., 2015)

405. Radius, distal condyle orientation in anterior view: perpendicular or beveled less than 15° to long axis of shaft (0); beveled at least 15° to long axis of shaft (1).

(Curry Rogers & Forster 2001; Wilson 2002, modified by Tschopp et al., 2015)

406. Radius, distal breadth: <1.8 times larger than midshaft breadth (0); at least 1.8 times midshaft breadth (1).

(Wilson 2002, modified by Tschopp et al., 2015)

407. Carpus, number of carpal bones: 3 or more (0); 2 (1); 1 or less (2).

(McIntosh 1990a; Upchurch 1998, modified by Tschopp et al., 2015)

408. Carpals: block-like (0); proximodistally compressed discs (1).

(Tschopp et al., 2015)

409. Metacarpus, shape: spreading (0); bound, with subparallel shafts and articular surfaces that extend half their length (1).

(Wilson 2002)

410. Metacarpals, shape of proximal surface in articulation: gently curving, forming a 90° arc (0); U-shaped, subtending a 270° arc (1).

(Wilson 2002)

411. Metacarpus, ratio of longest metacarpal to radius: < 0.40 (0); 0.40 or greater (1).

(McIntosh 1990, Calvo and Salgado 1995, modified by Mannion et al. 2013)

412. Metacarpal I, length: shorter than IV (0); longer than IV (1).

(Wilson & Sereno 1998, Wilson 2002)

413. Metacarpal I, proximal end dorsoventral height to mediolateral width ratio: < 1.8 (0); 1.8 or greater (1).

(Mannion et al., 2013, based on Apesteguía 2005, and Mannion & Calvo 2011)

414. Metacarpal III, robustness (length/distal transverse width): robust, <2.9 (0); intermediate, 2.9-3.5 (1); slender, > 3.5 (2).

(Tschopp et al., 2015)

415. Metacarpal V, proximal articular surface: subequal to smaller than (0); or significantly larger than proximal articular surface of mc III and IV (1).

(Tschopp et al., 2015)

416. Manual phalanx I-1, flange-like sheet of bone projecting from the proximoventral margin: absent (0); present (1).

(Hatcher 1901b, Gilmore 1936, Upchurch et al. 2004b)

417. Ilium, ratio of blade height above pubic peduncle to anteroposterior length: <0.40 (0); 0.40 or more (1).

(Tschopp et al., 2015)

418. Iliac preacetabular process, shape: sharply pointed (0); blunt to semicircular anterior margin (1).

(Salgado et al. 1997)

419. Ilium, preacetabular process, orientation of anterior tip in dorsal view: pointing anterolaterally (0); pointing laterally (1).

(Wilson 2002; wording modified by Tschopp et al., 2015)

420. Ilium, angle between the ventral edge of anterior iliac lobe and the anterior surface of the pubis process: is ~90° (0); is acute (1).

(Gilmore 1936, Upchurch et al. 2004b)

421. Ilium, dorsal margin shape: flat to slightly convex (0); semicircular (1).

(Wilson 2002, modified by Tschopp et al., 2015)

422. Ilium, highest point on dorsal margin: lies posterior to base of pubic process (0); lies anterior to base of pubic process (1).

(Upchurch et al. 2004a)

423. Ilium, pubic peduncle (measured at the articular surface), anteroposterior to mediolateral width ratio: > 0.80 (0); 0.80 or less (1).

(Mannion et al., 2013, modified by Tschopp et al., 2015)

424. Ilium, triangular fossa laterally at base of pubic peduncle: absent (0); present (1).

(Tschopp et al., 2015)

425. Ilium, distinct tubercle in the postacetabular region: absent (0); present (1).

(Carballido et al. 2012 (juvenile sauropod))

426. Pubis, ambiens process development: small, confluent not differentiated from the anterior border of the pubis (0); evident, but not especially developed (1); prominent, hook-like (2).

(McIntosh 1990a, Yu 1993, wording modified by Tschopp et al., 2015)

427. Pubis, length of puboischial contact: less than 0.41 total length of pubis (0); 0.41 or more of total length of pubis (1).

(Salgado et al. 1997, modified by Tschopp et al., 2015)

428. Pubis, participation in acetabulum: subequal to larger, compared to ischium (0); significantly smaller (1).

(Janensch 1961; Tschopp et al. 2015)

429. Ischium, acetabular articular surface: maintains approximately the same transverse width throughout its length (0); is transversely narrower in its central portion and strongly expanded as it approaches the iliac and pubic articulations (1).

(Mannion et al. 2012)

430. Ischium, acetabular margin, in lateral view: flat or mildly concave (0); strongly concave, pubic articular surface forms an anterodorsal projection (1).

(D'Emic 2012, modified by Mannion et al., 2013)

431. Ischium, iliac peduncle: iliac peduncle straight or widening in smooth curve distally (0); narrow, with distinct 'neck' (1).

(Sereno et al. 2007)

432. Ischia pubic articulation/anteroposterior length of pubic pedicel: < 1.5 (0); 1.5 or greater (1).

(Salgado et al., 1997)

433. Ischium, elongate muscle scar on proximal end: absent (0); present (1).

(Sereno et al. 2007)

434. Ischium, lateral fossa at base of shaft: absent (0); present (1).

(Wilson 2002)

435. Ischial distal shaft, shape: blade-like, medial and lateral depths subequal (0); triangular, depth of ischial shaft increases medially (1).

(Wilson 2002)

436. Ischial distal shafts, cross-sectional shape: V-shaped, forming an angle of nearly 50° with each other (0); flat, nearly coplanar (1).

(Upchurch 1998; Wilson & Sereno 1998)

437. Ischial shaft, transverse distal expansion: absent (0); present (1).

(Whitlock 2011)

438. Ischium, posterodorsal expansion of distal end: absent (0); present (1).

(Lovelace et al. 2007)

439. Femur, robustness index (sensu Wilson & Upchurch 2003): gracile, <0.22 (0); intermediate, 0.22-0.25 (1); robust, > 0.25 (2).

(Janensch 1961; Tschopp et al. 2015)

440. Femur, lateral bulge (marked by the lateral expansion and a dorsomedial orientation of the laterodorsal margin of the femur, which starts below the femur head ventral margin): absent (0); present (1).

(Salgado et al., 1997, modified by Tschopp et al., 2015)

441. Femoral shaft, lateral margin shape: straight (0); proximal one-third deflected medially (1).

(Wilson 2002)

442. Femur, cross-sectional shape: subequal to anteroposterior diameter (0); 125-150% anteroposterior diameter (1); at least 185% anteroposterior diameter (2).

(Wilson & Smith 1996; Tschopp et al. 2015)

443. Femoral head, position of highest point in anterior view: above point of maximum curvature of ventral edge of femoral head (0); laterally shifted, above main portion of shaft (1).

(Tschopp et al., 2015)

444. Femur, ventral surface of head: confluent with shaft (0); stepped (1).

(Tschopp et al., 2015)

445. Femur, greatest anteroposterior thickness of shaft: less than or approximately equal to half anteroposterior depth of distal articular condyles (0); much greater than half anteroposterior depth of distal articular condyles (1).

(Whitlock 2011)

446. Femur, large nutrient foramen opening midshaft anteriorly on femur: absent (0); present (1).

(Wilson 2002)

447. Femur, pronounced ridge on posterior surface between greater trochanter and head: absent (0); present (1).

(Sereno et al. 2007)

448. Femur, fourth trochanter: not visible in anterior view (0); prominent, visible in anterior view (1).

(Gallina & Apesteguía 2005, modified by Whitlock 2011)

449. Femoral fourth trochanter, present as low rounded ridge (0); greatly reduced so that it is virtually absent (1).

(Mannion et al. 2012)

450. Femur, fourth trochanter, position: distally displaced (0); on proximal half of shaft (1).

((Schwarz-Wings & Böhm, 2014); Tschopp et al., 2015)

451. Femur, shape of distal condyles: articular surface restricted to distal portion of femur (0); expanded onto anterior portion of femoral shaft (1).

(Wilson & Carrano 1999; Wilson 2002)

452. Tibia to femur length: < 0.68 (0); 0.68 or greater (1).

(Gauthier 1986; Upchurch 1998)

453. Tibia, proximal articulation surface, shape: subcircular to transversely compressed (0); anteroposteriorly compressed (1).

(Wilson 2002, modified by Tschopp et al., 2015)

454. Tibia, proximal articular surface, shape: subrectangular (0); subtriangular (1).

((Harris & Dodson 2004); Tschopp et al., 2015)

455. Tibia, short transverse ridge on anteromedial surface of distal end: absent (0); present (1).

(Tschopp et al., 2015)

456. Tibia, cnemial crest in anterior view: widely rounded (0); subtriangular (1).

(Tschopp et al., 2015)

457. Tibia, posterior surface of cnemial crest: smooth (0); bears a distinct fibular trochanter (1).

(Harris (2007))

458. Tibia, lateral edge of proximal end forms a pinched out projection, posterior to cnemial crest (the 'second cnemial crest' of Bonaparte et al., 2000): present (0); absent (1).

(Mannion et al., 2013)

459. Fibula, proximal end with anteromedially directed crest extending into a notch behind the cnemial crest of the tibia: absent (0); present (1).

(Wilson & Upchurch 2009; D'Emic 2012, modified by Mannion et al., 2013)

460. Fibula, insertion of the M. iliofibularis: located approximately at mid-shaft (0); proximal, located above midshaft (1).

(Whitlock 2011)

461. Astragalus, morphology in anterior view: rectangular (0); wedge-shaped, narrowing medially (1).

(Upchurch 1995, 1998, modified by Nair & Salisbury (2012))

462. Astragalus, anteroposterior dimension as seen in dorsal view: widens medially or does not change in width (0); narrows medially (1).

(Cooper 1984, Upchurch 1998)

463. Astragalus, proximodistal length/transverse breadth: < 0,55 (0); 0,55 or greater (1).

((McIntosh et al. 1992); Tschopp et al., 2015)

464. Astragalus, mediolateral width to maximum anteroposterior length ratio: 1.6 or greater (0); < 1.6 (1).

(Sander et al. 2006, modified by Tschopp et al., 2015)

465. Astragalus, ascending process length: limited to anterior two-thirds of astragalus anteroposterior width (0); extends beyond two-thirds of astragalus anteroposterior width (normally to posterior margin of astragalus) (1).

(Wilson & Sereno 1998, Wilson 2002, modified by Mannion et al. 2012)

466. Astragalus, fibular facet: faces laterally (0); faces posterolaterally, anterior margin visible in posterior view (1).

(Whitlock 2011)

467. Astragalus, laterally directed ventral shelf underlies distal end of fibula: present (0); absent (1).

(Mannion et al., 2013, based on Wilson & Upchurch 2009)

468. Astragalus, dorsomedial corner in posterior view: short and blunt (0); elongate and narrow (1).

(Tschopp et al., 2015)

469. Calcaneum: proximodistally compressed (0); globular (1).

(Harris & Dodson 2004)

470. Metatarsals, metatarsal I to metatarsal V proximodistal length ratio: 1.0 or greater (0); < 1.0 (1).

(Mannion et al., 2013, polarity reversed by Tschopp et al., 2015)

471. Metatarsal I, dorsal/anterior surface: without foramina (0); several foramina present (1).

(Tschopp et al., 2015)

472. Metatarsal I proximal articular surface, transverse axis orientation: angled ventromedially approximately 15º to (0); perpendicular to axis of shaft (1).

(Wilson 2002, modified by Carballido et al., 2012, polarity reversed by Tschopp et al., 2015)

473. Metatarsal I, robustness (proximal transverse width/greatest length): relatively gracile, < 0.8 (0); robust, 0.8 or more (1).

(Upchurch et al. 2004a, modified by Tschopp et al., 2015)

474. Metatarsal I distal articular surface, transverse axis orientation: angled dorsomedially to (0); perpendicular to axis of shaft (1).

(Wilson 2002, modified by Carballido et al., 2012, polarity reversed by Tschopp et al., 2015)

475. Metatarsal I distal condyle, posterolateral projection: absent (0); present (1).

((Berman & McIntosh 1978); Tschopp et al., 2015)

476. Metatarsal I, distolateral projection, if present: small and blunt, not projecting considerably lateral to dorsal edge of distal articular surface (0); prominent and pointed, reaching significantly more laterally than dorsal edge of distal articular surface (1).

((McIntosh 1990); Tschopp et al., 2015)

477. Metatarsals I-III, rugosities on dorsolateral margins near distal ends: absent (0); present (1).

(Upchurch 1995)

478. Metatarsal II, robustness (mean transverse breadth proximal and distal/maximum length): slender, <0.53 (0); intermediate, 0.53-0.65 (1); robust, >0.65 (2).

(Tschopp et al., 2015)

479. Metatarsal II, lateral margin in proximal view: concave (0); straight (1).

(Mannion et al. 2013)

480. Metatarsal II, rugosity on dorsolateral margin near distal end (if present): shallow (0); well-developed, extending to center of shaft (1).

(Tschopp et al., 2015)

481. Metatarsal II distal condyle, posterolateral projection: absent (0); present (1).

(Tschopp et al., 2015)

482. Metatarsal IV, proximal articular surface, outline: L- to V-shaped, with distinctly concave posterolateral edge (0); subtriangular (1).

(Tschopp et al., 2015)

483. Metatarsal V, proximal articular surface, shape: triangular (0); rhomboid (1).

(Tschopp et al., 2015)

484. Metatarsal V proximal end to distal end maximum mediolateral width ratio: 1.6 or greater (0); < 1.6 (1).

(Mannion et al., 2013)

485. Pes, phalanx I-1: proximal and ventral surfaces meet at approximately 90° (0); proximoventral corner drawn out into thin plate underlying metatarsal I (1).

((McIntosh et al. 1992); Tschopp et al., 2015)

486. Pes, phalanx I-1, distal articular surface shape: wide, maximum transverse width > 1.1 times anteroposterior height (0); narrow, maximum transverse width 1.1 times anteroposterior height or less (1).

(Tschopp et al., 2015)

487. Pes, phalanx II-2: well developed and subrectangular in dorsal view (0); reduced, with an irregular D-shaped outline and proximal and distal articular surfaces that meet virtually along dorsal and plantar margins (1).

((McIntosh et al. 1992); Tschopp et al., 2015)

488. Pes, phalanges III-1 and IV-1: equal to longer than wide (0); wider than long (1).

((McIntosh et al. 1992); Tschopp et al., 2015)

489. Pedal unguals, groove on lateral surface: follows curvature of claw (0); straight horizontally (1).

(Tschopp et al., 2015)
