# Supplementary material for: Osteology of Galeamopus pabsti sp. nov. (Sauropoda: Diplodocidae), with implications for neurocentral closure timing, and the cervico-dorsal transition in diplodocids
Source: PeerJ. 2017 May 2;5:e3179. doi: 10.7717/peerj.3179 (PMC5417106; doi:10.7717/peerj.3179)
Supplement: Data S2 — List of the autapomorphies and synapomorphies of the OTUs and nodes of the phylogenetic analysis. [file peerj-05-3179-s002.docx]

# Osteology of*Galeamopus pabsti*sp. nov. (Sauropoda: Diplodocidae), with implications on neurocentral closure timing, and cervico-dorsal transition in diplodocids

# Supplementary Files

Phylogenetic analysis

# Apomorphies recovered by TNT

Note that the character numbers are the ones provided by TNT, and start with character 0.

## Autapomorphies

*Shunosaurus lii* :

No autapomorphies

*Omeisaurus* :

Char. 75: 0 --> 1

Char. 79: 0 --> 1

Char. 169: 0 --> 2

Char. 175: 0 --> 1

Char. 183: 0 --> 1

Char. 203: 0 --> 1

Char. 220: 0 --> 1

Char. 337: 0 --> 1

Char. 352: 0 --> 1

Char. 376: 1 --> 0

Char. 381: 0 --> 1

Char. 384: 0 --> 1

Char. 390: 1 --> 2

Char. 398: 1 --> 2

Char. 400: 1 --> 0

Char. 404: 0 --> 1

Char. 425: 0 --> 1

Char. 487: 1 --> 0

*Mamenchisaurus* :

Char. 4: 1 --> 0

Char. 53: 0 --> 1

Char. 60: 0 --> 1

Char. 76: 0 --> 1

Char. 128: 0 --> 1

Char. 133: 0 --> 1

Char. 234: 0 --> 1

Char. 255: 0 --> 1

Char. 282: 0 --> 1

Char. 304: 1 --> 0

Char. 313: 0 --> 3

Char. 374: 1 --> 0

Char. 380: 0 --> 1

Char. 410: 0 --> 1

Char. 477: 1 --> 0

Char. 486: 0 --> 1

*Jobaria tiguidensis* :

Char. 4: 1 --> 0

Char. 75: 0 --> 1

Char. 144: 0 --> 1

Char. 160: 1 --> 0

Char. 163: 0 --> 1

Char. 183: 0 --> 1

Char. 188: 0 --> 1

Char. 201: 0 --> 1

Char. 203: 0 --> 1

Char. 218: 0 --> 1

Char. 253: 0 --> 1

Char. 308: 1 --> 0

Char. 358: 1 --> 0

Char. 362: 0 --> 1

Char. 363: 0 --> 1

Char. 386: 0 --> 1

Char. 404: 0 --> 1

Char. 423: 0 --> 1

Char. 444: 0 --> 1

Char. 456: 0 --> 1

Char. 467: 0 --> 1

*Camarasaurus* :

Char. 8: 0 --> 1

Char. 37: 1 --> 0

Char. 71: 0 --> 1

Char. 74: 1 --> 0

Char. 75: 0 --> 1

Char. 103: 0 --> 1

Char. 128: 0 --> 1

Char. 130: 1 --> 0

Char. 138: 0 --> 1

Char. 146: 1 --> 0

Char. 155: 0 --> 1

Char. 156: 1 --> 0

Char. 157: 1 --> 2

Char. 162: 0 --> 1

Char. 178: 0 --> 1

Char. 206: 1 --> 0

Char. 240: 0 --> 1

Char. 253: 0 --> 1

Char. 256: 0 --> 1

Char. 264: 1 --> 0

Char. 268: 0 --> 1

Char. 280: 0 --> 1

Char. 290: 0 --> 1

Char. 294: 01 --> 2

Char. 326: 0 --> 1

Char. 336: 0 --> 1

Char. 337: 0 --> 1

Char. 348: 1 --> 0

Char. 358: 1 --> 0

Char. 363: 0 --> 1

Char. 374: 1 --> 0

Char. 384: 0 --> 1

Char. 386: 0 --> 1

Char. 398: 1 --> 2

Char. 402: 0 --> 1

Char. 420: 0 --> 1

Char. 438: 1 --> 2

Char. 444: 0 --> 1

Char. 467: 0 --> 1

Char. 472: 1 --> 0

*Giraffatitan brancai* :

Char. 48: 0 --> 1

Char. 65: 1 --> 0

Char. 81: 0 --> 1

Char. 85: 1 --> 0

Char. 169: 0 --> 2

Char. 240: 0 --> 1

Char. 244: 0 --> 1

Char. 259: 0 --> 1

Char. 262: 1 --> 0

Char. 268: 0 --> 1

Char. 273: 0 --> 1

Char. 288: 1 --> 0

Char. 315: 0 --> 1

Char. 322: 0 --> 1

Char. 383: 0 --> 1

Char. 384: 0 --> 1

Char. 450: 0 --> 1

*Brachiosaurus altithorax* :

Char. 27: 0 --> 1

Char. 32: 0 --> 1

Char. 87: 0 --> 2

Char. 164: 1 --> 0

Char. 294: 1 --> 2

Char. 336: 0 --> 1

Char. 416: 0 --> 1

Char. 419: 0 --> 1

Char. 424: 0 --> 1

Char. 443: 1 --> 0

Char. 445: 0 --> 1

*Ligabuesaurus leanzai* :

Char. 130: 1 --> 2

Char. 144: 0 --> 1

Char. 249: 0 --> 1

Char. 373: 0 --> 1

Char. 377: 1 --> 0

Char. 380: 0 --> 1

*Isisaurus colberti* :

Char. 174: 1 --> 0

Char. 242: 0 --> 1

Char. 275: 1 --> 0

Char. 368: 0 --> 1

*Haplocanthosaurus priscus* :

Char. 138: 0 --> 1

Char. 141: 1 --> 0

Char. 162: 0 --> 1

Char. 163: 0 --> 1

Char. 170: 0 --> 1

Char. 178: 0 --> 1

Char. 181: 0 --> 1

Char. 192: 0 --> 1

Char. 230: 0 --> 1

Char. 243: 0 --> 1

Char. 248: 0 --> 1

Char. 306: 0 --> 1

Char. 333: 0 --> 1

Char. 377: 1 --> 0

Char. 384: 0 --> 1

Char. 417: 0 --> 1

Char. 423: 0 --> 1

Char. 432: 0 --> 1

*Limaysaurus tessonei* :

Char. 192: 0 --> 1

Char. 306: 0 --> 1

Char. 315: 0 --> 1

Char. 359: 0 --> 1

*Cathartesaura anaerobica* :

Char. 204: 0 --> 1

Char. 330: 0 --> 1

Char. 348: 1 --> 0

Char. 352: 0 --> 1

*Zapalasaurus bonapartei* :

Char. 318: 0 --> 1

Char. 402: 0 --> 1

*Nigersaurus taqueti* :

Char. 109: 0 --> 1

Char. 114: 0 --> 1

Char. 116: 1 --> 0

Char. 169: 0 --> 1

Char. 175: 0 --> 1

Char. 187: 0 --> 1

Char. 190: 0 --> 1

Char. 219: 1 --> 0

Char. 226: 0 --> 1

Char. 244: 0 --> 1

Char. 337: 0 --> 1

*Demandasaurus darwini* :

Char. 116: 1 --> 2

Char. 121: 1 --> 0

Char. 199: 0 --> 1

Char. 282: 0 --> 1

Char. 291: 1 --> 2

Char. 334: 1 --> 0

Char. 336: 0 --> 1

Char. 339: 0 --> 1

Char. 342: 0 --> 1

Char. 343: 2 --> 3

Char. 441: 1 --> 2

*Dicraeosaurus hansemanni* :

Char. 32: 0 --> 1

Char. 34: 0 --> 1

Char. 48: 0 --> 1

Char. 62: 1 --> 0

Char. 100: 1 --> 0

Char. 155: 0 --> 1

Char. 164: 0 --> 1

Char. 174: 1 --> 0

Char. 213: 0 --> 1

Char. 218: 0 --> 1

Char. 222: 0 --> 1

Char. 229: 1 --> 0

Char. 271: 0 --> 1

Char. 441: 1 --> 0

Char. 450: 0 --> 1

Char. 455: 1 --> 0

*Brachytrachelopan mesai* :

Char. 145: 0 --> 1

Char. 183: 0 --> 1

Char. 194: 0 --> 1

Char. 203: 0 --> 1

Char. 264: 1 --> 0

*Amargasaurus cazaui* :

Char. 187: 0 --> 1

Char. 188: 0 --> 1

Char. 192: 0 --> 1

Char. 212: 1 --> 0

Char. 215: 1 --> 0

Char. 249: 0 --> 1

Char. 250: 1 --> 0

Char. 261: 0 --> 1

Char. 290: 0 --> 1

*Suuwassea emilieae* :

Char. 87: 01 --> 2

Char. 102: 0 --> 1

Char. 116: 1 --> 2

Char. 144: 0 --> 1

Char. 145: 0 --> 1

Char. 148: 0 --> 1

Char. 159: 0 --> 1

Char. 161: 0 --> 1

Char. 169: 0 --> 1

Char. 178: 0 --> 1

Char. 182: 0 --> 1

Char. 183: 0 --> 1

Char. 187: 0 --> 1

Char. 195: 0 --> 1

Char. 223: 0 --> 1

Char. 230: 0 --> 1

Char. 248: 0 --> 1

Char. 249: 0 --> 1

Char. 305: 0 --> 1

Char. 318: 0 --> 1

Char. 391: 1 --> 2

Char. 470: 0 --> 1

*Amphicoelias altus* :

Char. 262: 0 --> 1

Char. 277: 1 --> 0

Char. 284: 1 --> 0

Char. 438: 1 --> 0

*Apatosaurus ajax* :

Char. 54: 0 --> 1

Char. 83: 0 --> 1

Char. 87: 1 --> 2

Char. 89: 1 --> 0

Char. 145: 0 --> 1

Char. 171: 1 --> 0

Char. 211: 0 --> 1

Char. 249: 0 --> 1

Char. 264: 1 --> 0

Char. 267: 0 --> 1

Char. 278: 0 --> 1

Char. 281: 1 --> 0

Char. 301: 0 --> 1

Char. 370: 1 --> 0

Char. 371: 1 --> 2

Char. 379: 0 --> 1

Char. 437: 0 --> 1

*Apatosaurus louisae* :

Char. 168: 0 --> 1

Char. 199: 0 --> 1

Char. 204: 1 --> 0

Char. 222: 0 --> 1

Char. 223: 0 --> 1

Char. 224: 0 --> 1

Char. 227: 0 --> 1

Char. 231: 1 --> 0

Char. 246: 0 --> 1

Char. 247: 1 --> 0

Char. 253: 0 --> 1

Char. 292: 0 --> 1

Char. 293: 0 --> 1

Char. 297: 1 --> 0

Char. 306: 0 --> 1

Char. 319: 1 --> 0

Char. 326: 1 --> 0

Char. 335: 0 --> 1

Char. 336: 1 --> 0

Char. 380: 0 --> 1

Char. 399: 1 --> 0

*Brontosaurus excelsus* :

Char. 141: 1 --> 0

Char. 215: 1 --> 0

Char. 222: 0 --> 1

Char. 278: 0 --> 1

Char. 293: 0 --> 1

Char. 352: 0 --> 1

Char. 370: 1 --> 0

Char. 379: 0 --> 1

Char. 416: 0 --> 1

Char. 421: 0 --> 1

Char. 445: 0 --> 1

Char. 466: 0 --> 1

*Brontosaurus yahnahpin* :

Char. 130: 1 --> 2

Char. 156: 1 --> 0

Char. 164: 0 --> 1

Char. 223: 0 --> 1

Char. 251: 1 --> 0

Char. 319: 1 --> 0

Char. 330: 1 --> 0

Char. 369: 1 --> 0

Char. 383: 1 --> 0

Char. 399: 1 --> 0

Char. 405: 1 --> 0

Char. 410: 0 --> 1

*Brontosaurus parvus* :

Char. 144: 0 --> 1

Char. 156: 1 --> 2

Char. 162: 0 --> 1

Char. 244: 1 --> 0

Char. 248: 0 --> 1

Char. 250: 0 --> 1

Char. 260: 0 --> 1

Char. 266: 0 --> 1

Char. 278: 0 --> 1

Char. 283: 1 --> 0

Char. 314: 1 --> 0

*Tornieria africana* :

Char. 81: 0 --> 1

Char. 169: 1 --> 2

Char. 177: 0 --> 1

Char. 178: 0 --> 1

Char. 190: 1 --> 0

Char. 342: 1 --> 0

Char. 345: 0 --> 1

Char. 371: 1 --> 0

Char. 388: 0 --> 1

Char. 437: 0 --> 1

Char. 443: 1 --> 0

Char. 450: 0 --> 1

*Supersaurus vivianae* :

Char. 134: 1 --> 0

Char. 139: 0 --> 1

Char. 176: 0 --> 1

Char. 237: 1 --> 0

Char. 260: 1 --> 0

Char. 265: 0 --> 1

Char. 269: 0 --> 1

Char. 305: 0 --> 1

Char. 313: 2 --> 1

Char. 316: 1 --> 0

*Supersaurus lourinhanensis* :

Char. 314: 1 --> 0

*Leinkupal laticauda* :

Char. 319: 1 --> 0

Char. 323: 1 --> 0

*Galeamopus hayi* :

Char. 65: 1 --> 0

Char. 67: 1 --> 0

Char. 76: 0 --> 1

Char. 100: 1 --> 0

Char. 145: 0 --> 1

Char. 169: 1 --> 0

Char. 210: 1 --> 0

Char. 398: 1 --> 2

Char. 403: 1 --> 0

Char. 482: 0 --> 1

*Galeamopus pabsti* :

Char. 83: 0 --> 1

Char. 87: 1 --> 2

Char. 137: 0 --> 1

Char. 157: 1 --> 2

Char. 170: 0 --> 1

Char. 186: 1 --> 0

Char. 187: 1 --> 0

Char. 191: 0 --> 1

Char. 196: 0 --> 1

Char. 197: 1 --> 0

Char. 207: 0 --> 1

Char. 253: 0 --> 1

Char. 369: 1 --> 2

Char. 391: 1 --> 2

Char. 402: 0 --> 1

Char. 410: 0 --> 1

Char. 414: 0 --> 1

Char. 453: 1 --> 0

*Diplodocus carnegii* :

Char. 253: 0 --> 1

Char. 286: 0 --> 1

Char. 304: 0 --> 1

Char. 432: 1 --> 0

Char. 477: 1 --> 0

*Diplodocus hallorum* :

Char. 203: 1 --> 0

Char. 210: 1 --> 0

Char. 212: 1 --> 0

Char. 240: 0 --> 1

Char. 259: 1 --> 0

Char. 260: 1 --> 0

Char. 346: 0 --> 1

Char. 349: 0 --> 1

Char. 352: 0 --> 1

Char. 366: 0 --> 1

Char. 437: 0 --> 1

Char. 438: 1 --> 0

Char. 488: 0 --> 1

*Kaatedocus siberi* :

Char. 2: 1 --> 0

Char. 12: 1 --> 0

Char. 28: 1 --> 0

Char. 33: 0 --> 1

Char. 50: 0 --> 1

Char. 54: 0 --> 1

Char. 57: 1 --> 0

Char. 76: 0 --> 1

Char. 77: 0 --> 1

Char. 85: 1 --> 2

Char. 98: 0 --> 1

Char. 146: 1 --> 0

Char. 160: 1 --> 0

Char. 182: 0 --> 1

Char. 200: 0 --> 1

Char. 201: 0 --> 1

Char. 207: 0 --> 1

Char. 216: 0 --> 1

Char. 217: 0 --> 1

*Barosaurus lentus* :

Char. 140: 0 --> 1

Char. 169: 1 --> 2

Char. 176: 0 --> 1

Char. 192: 0 --> 1

Char. 197: 1 --> 2

Char. 205: 0 --> 1

Char. 218: 0 --> 1

Char. 229: 2 --> 3

Char. 248: 1 --> 0

Char. 311: 0 --> 1

Char. 330: 1 --> 0

Char. 371: 1 --> 0

Char. 391: 1 --> 0

Char. 442: 0 --> 1

Clade Synapomorphies

*Mamenchisaurus* + *Diplodocus*:

No synapomorphies

Mamenchisauridae:

Char. 32: 0 --> 1

Char. 129: 0 --> 2

Char. 136: 0 --> 1

Char. 138: 0 --> 1

Char. 157: 1 --> 0

Char. 158: 0 --> 1

Char. 254: 0 --> 1

Char. 272: 0 --> 1

Char. 275: 1 --> 0

Char. 279: 01 --> 2

Char. 419: 0 --> 1

Char. 443: 1 --> 0

Char. 472: 1 --> 0

*Jobaria tiguidensis* + Neosauropoda :

Char. 35: 0 --> 1

Char. 85: 0 --> 1

Char. 156: 0 --> 1

Char. 164: 0 --> 1

Char. 262: 0 --> 1

Char. 264: 0 --> 1

Char. 329: 0 --> 1

Char. 332: 0 --> 1

Char. 348: 0 --> 1

Char. 377: 0 --> 1

Char. 378: 0 --> 1

Char. 408: 0 --> 1

Char. 435: 0 --> 1

Char. 460: 0 --> 1

Char. 474: 1 --> 0

Neosauropoda :

Char. 5: 0 --> 1

Char. 116: 0 --> 1

Char. 127: 0 --> 1

Char. 283: 0 --> 1

Char. 353: 0 --> 1

Char. 385: 0 --> 2

Char. 397: 0 --> 1

Char. 403: 1 --> 0

Char. 406: 0 --> 1

Macronaria:

Char. 67: 0 --> 1

Char. 93: 0 --> 1

Char. 278: 0 --> 1

Char. 279: 1 --> 2

Char. 395: 0 --> 1

Char. 410: 0 --> 1

Char. 412: 0 --> 1

Char. 426: 0 --> 1

Char. 427: 1 --> 0

Char. 439: 0 --> 1

Char. 449: 0 --> 1

Char. 452: 1 --> 0

Char. 454: 0 --> 1

Char. 466: 0 --> 1

Char. 468: 1 --> 0

Titanosauriformes :

Char. 133: 0 --> 1

Char. 343: 0 --> 1

Char. 346: 0 --> 1

Char. 362: 0 --> 1

Char. 375: 0 --> 1

Char. 378: 1 --> 0

Char. 381: 0 --> 1

Char. 390: 1 --> 3

Char. 391: 1 --> 0

Char. 398: 1 --> 0

Char. 417: 0 --> 1

Char. 421: 0 --> 1

Char. 440: 0 --> 1

Char. 441: 1 --> 2

Brachiosauridae :

Char. 136: 0 --> 1

Char. 187: 0 --> 1

Char. 270: 0 --> 1

Somphospondyli :

Char. 141: 1 --> 0

Char. 234: 0 --> 1

Char. 239: 0 --> 1

Char. 283: 1 --> 0

Char. 376: 1 --> 0

Diplodocoidea :

Char. 167: 1 --> 0

Char. 219: 0 --> 1

Char. 220: 0 --> 1

Char. 270: 0 --> 1

Char. 297: 0 --> 1

Char. 465: 0 --> 1

Diplodocimorpha :

Char. 164: 1 --> 0

Char. 206: 1 --> 0

Char. 275: 1 --> 0

Char. 281: 0 --> 1

Char. 291: 0 --> 1

Char. 309: 0 --> 1

Char. 326: 0 --> 1

Char. 327: 0 --> 1

Char. 420: 0 --> 1

Rebbachisauridae :

Char. 202: 0 --> 1

Char. 321: 0 --> 1

Char. 334: 0 --> 1

Char. 343: 0 --> 2

Char. 471: 0 --> 1

Char. 473: 0 --> 1

*Nigersaurus* + *Limaysaurus* :

Char. 345: 0 --> 1

Char. 428: 0 --> 1

Char. 430: 0 --> 1

Char. 432: 0 --> 1

Limaysaurinae:

Char. 328: 0 --> 1

Char. 373: 0 --> 1

Nigersaurinae :

Char. 269: 0 --> 1

Char. 301: 0 --> 1

Char. 446: 0 --> 1

Flagellicaudata :

Char. 9: 0 --> 1

Char. 16: 0 --> 1

Char. 56: 0 --> 1

Char. 58: 0 --> 1

Char. 60: 0 --> 1

Char. 61: 0 --> 1

Char. 89: 0 --> 1

Char. 106: 0 --> 1

Char. 124: 0 --> 1

Char. 128: 0 --> 1

Char. 268: 0 --> 1

Char. 285: 1 --> 0

Char. 309: 1 --> 3

Char. 312: 0 --> 1

Char. 314: 0 --> 1

Char. 336: 0 --> 1

Char. 348: 1 --> 0

Char. 361: 1 --> 0

Char. 381: 0 --> 1

Char. 425: 0 --> 1

Char. 434: 0 --> 1

Char. 435: 1 --> 0

Char. 436: 0 --> 1

Char. 455: 0 --> 1

Char. 474: 0 --> 1

Char. 476: 0 --> 1

Char. 480: 0 --> 1

Dicraeosauridae :

Char. 37: 1 --> 0

Char. 76: 0 --> 1

Char. 77: 0 --> 1

Char. 85: 1 --> 0

Char. 107: 0 --> 1

Char. 108: 0 --> 1

Char. 114: 0 --> 1

Char. 130: 1 --> 0

Char. 160: 1 --> 0

Char. 163: 0 --> 1

Char. 172: 0 --> 1

Char. 216: 0 --> 1

Char. 250: 0 --> 1

Char. 472: 1 --> 0

*Dicraeosaurus* + *Amargasaurus*:

Char. 27: 0 --> 1

Char. 35: 1 --> 0

Char. 73: 0 --> 1

Char. 74: 1 --> 0

Char. 132: 1 --> 0

Char. 135: 1 --> 0

Char. 173: 0 --> 1

Char. 233: 1 --> 0

Char. 397: 1 --> 0

*Amargasaurus* + *Brachytrachelopan*:

Char. 141: 1 --> 0

Char. 181: 0 --> 1

Char. 185: 1 --> 0

Char. 232: 1 --> 0

Char. 252: 0 --> 1

Char. 279: 1 --> 0

Char. 287: 1 --> 0

Diplodocidae :

Char. 26: 0 --> 1

Char. 52: 0 --> 1

Char. 129: 0 --> 1

Char. 190: 0 --> 1

Char. 204: 0 --> 1

Char. 229: 1 --> 2

Char. 270: 1 --> 0

Char. 319: 0 --> 1

Char. 323: 0 --> 1

Char. 328: 0 --> 1

Char. 330: 0 --> 1

Char. 403: 0 --> 1

Apatosaurinae :

Char. 138: 0 --> 1

Char. 176: 0 --> 1

Char. 178: 0 --> 1

Char. 191: 0 --> 1

Char. 221: 0 --> 1

Char. 225: 0 --> 1

Char. 228: 0 --> 1

Char. 260: 1 --> 0

Char. 262: 1 --> 0

Char. 305: 0 --> 1

Char. 375: 0 --> 1

Char. 383: 0 --> 1

Char. 391: 1 --> 2

Char. 402: 0 --> 1

Char. 406: 1 --> 2

Char. 407: 0 --> 1

Char. 411: 0 --> 1

Char. 452: 1 --> 0

*Apatosaurus* :

Char. 172: 0 --> 1

Char. 188: 0 --> 1

Char. 192: 0 --> 1

Char. 378: 1 --> 0

*Brontosaurus* :

Char. 206: 0 --> 1

Char. 243: 0 --> 1

Char. 279: 1 --> 0

Char. 302: 0 --> 1

Char. 338: 0 --> 1

Char. 359: 0 --> 1

*Amphicoelias altus* + *Brontosaurus yahnahpin*:

Char. 281: 1 --> 0

Char. 441: 1 --> 0

*Brontosaurus parvus* + *B. yahnahpin* :

Char. 275: 0 --> 1

Char. 280: 0 --> 1

Char. 282: 0 --> 1

Diplodocinae :

Char. 20: 0 --> 1

Char. 48: 0 --> 1

Char. 67: 0 --> 1

Char. 69: 0 --> 1

Char. 84: 0 --> 1

Char. 91: 0 --> 1

Char. 93: 0 --> 1

Char. 137: 1 --> 0

Char. 168: 0 --> 1

Char. 169: 0 --> 1

Char. 175: 0 --> 1

Char. 183: 0 --> 1

Char. 184: 0 --> 1

Char. 187: 0 --> 1

Char. 195: 0 --> 1

Char. 209: 0 --> 1

Char. 219: 1 --> 0

Char. 223: 0 --> 1

Char. 226: 0 --> 1

*Tornieria africana* + *Diplodocus*:

Char. 36: 0 --> 1

Char. 134: 0 --> 1

*Supersaurus* + *Diplodocus* :

Char. 348: 0 --> 1

Char. 423: 0 --> 1

Char. 431: 1 --> 0

Char. 459: 0 --> 1

*Supersaurus* :

Char. 180: 0 --> 1

Char. 181: 0 --> 1

Char. 257: 0 --> 1

Char. 277: 1 --> 0

Char. 293: 0 --> 1

*Leinkupal* + *Diplodocus* :

Char. 324: 0 --> 1

*Galeamopus* + *Diplodocus* :

Char. 307: 0 --> 1

*Galeamopus* :

Char. 139: 0 --> 1

Char. 195: 1 --> 0

Char. 419: 1 --> 0

Char. 457: 1 --> 0

*Barosaurus* + *Diplodocus* :

Char. 201: 0 --> 1

Char. 219: 0 --> 1

Char. 265: 0 --> 1

Char. 342: 1 --> 0

Char. 344: 0 --> 1

Char. 392: 0 --> 1

Char. 472: 1 --> 0

*Diplodocus* :

Char. 137: 0 --> 1

Char. 186: 1 --> 0

Char. 187: 1 --> 0

Char. 214: 1 --> 0

Char. 289: 0 --> 1

Char. 309: 3 --> 4

Char. 317: 0 --> 1

Char. 340: 0 --> 1

Char. 345: 0 --> 1

Char. 358: 3 --> 4

Char. 425: 1 --> 2

Char. 479: 0 --> 1
